# Vitronectin-GM-CSF fusion protein hydrogel with a recruitment-anchoring-activation strategy accelerates vascularized tissue regeneration

**DOI:** 10.1093/rb/rbag100

**Published:** 2026-05-23

**Authors:** Jiake Zhang, Yuhan Xia, Xueliang Peng, Wenjing Li, Yuzhen Zhao, Fulin Chen, Zhuoyue Chen

**Affiliations:** Provincial Key Laboratory of Biotechnology of Shaanxi, Key Laboratory of Resource Biology and Modern Biotechnology in Western China, Faculty of Life Sciences, Northwest University, 229 North Taibai Road, Xi’an 710069, Shaanxi Province, China; Provincial Key Laboratory of Biotechnology of Shaanxi, Key Laboratory of Resource Biology and Modern Biotechnology in Western China, Faculty of Life Sciences, Northwest University, 229 North Taibai Road, Xi’an 710069, Shaanxi Province, China; Provincial Key Laboratory of Biotechnology of Shaanxi, Key Laboratory of Resource Biology and Modern Biotechnology in Western China, Faculty of Life Sciences, Northwest University, 229 North Taibai Road, Xi’an 710069, Shaanxi Province, China; Provincial Key Laboratory of Biotechnology of Shaanxi, Key Laboratory of Resource Biology and Modern Biotechnology in Western China, Faculty of Life Sciences, Northwest University, 229 North Taibai Road, Xi’an 710069, Shaanxi Province, China; Provincial Key Laboratory of Biotechnology of Shaanxi, Key Laboratory of Resource Biology and Modern Biotechnology in Western China, Faculty of Life Sciences, Northwest University, 229 North Taibai Road, Xi’an 710069, Shaanxi Province, China; Provincial Key Laboratory of Biotechnology of Shaanxi, Key Laboratory of Resource Biology and Modern Biotechnology in Western China, Faculty of Life Sciences, Northwest University, 229 North Taibai Road, Xi’an 710069, Shaanxi Province, China; Provincial Key Laboratory of Biotechnology of Shaanxi, Key Laboratory of Resource Biology and Modern Biotechnology in Western China, Faculty of Life Sciences, Northwest University, 229 North Taibai Road, Xi’an 710069, Shaanxi Province, China

**Keywords:** vitronectin-GM-CSF fusion protein, VGC hydrogel, angiogenesis, immune modulation, tissue regeneration

## Abstract

The process of tissue injury repair involves the synergistic effects of angiogenesis and immune regulation, yet a single molecule rarely can regulate both processes. To address this, we designed a fusion protein, VGC, by integrating the potent chemotactic property of granulocyte-macrophage colony-stimulating factor (GM-CSF) with the cell-anchoring domain of vitronectin (VTN). This design yielded an integrated system capable of recruiting, anchoring and activating repair cells. The VGC, connecting VTN and GM-CSF via linker peptides (GGGGSGGGGS), was successfully produced by microbial fermentation and loaded into temperature-sensitive Pluronic F127 hydrogel (F127) for controlled release. *In vitro*, VGC significantly promoted endothelial cell proliferation, migration and tube formation, while temporally modulated macrophage polarization from the M1 to M2 phenotype to improve the immune microenvironment. In a rat cranial defect model, VGC-loaded hydrogel (F-VGC) accelerated bone regeneration, increasing bone volume (**P < 0.01, vs F-VTN; ***P < 0.01, vs F127) and collagen I deposition (*P < 0.05, vs F127). In mouse full-thickness skin wounds, F-VGC significantly accelerated healing, achieving a 95.78 ± 0.65% closure rate by Day 10 (**P < 0.01, vs F-VTN/F127) through mechanisms involving early angiogenesis, collagen remodeling and re-epithelialization. This study provides a protein-based strategy for synergistically promoting vascularization and immune modulation, offering a new paradigm for functional biomaterial design.

## Graphical Abstract

**Graphical Abstract:**
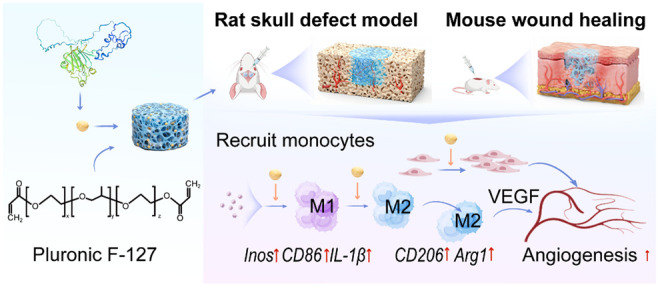

## Introduction

Tissue injury repair is a physiological process regulated by the coordinated action of multiple molecules, involving interactions between immune cells and parenchymal cells, with the synergy between immune regulation and angiogenesis being key to determining the effectiveness of the repair [1]. Whether the tissue defect is caused by trauma or inflammatory injury, it disrupts the homeostasis of the local microenvironment and the host’s inherent regenerative capacity is often limited, making it difficult to achieve complete structural and functional reconstruction for defects exceeding the critical size [2]. When endogenous repair capacity is insufficient, it often manifests as delayed inflammation resolution and slow angiogenesis, eventually leading to scar formation or functional decline [3–5]. Therefore, developing bioactive materials that can promote *in situ* immunomodulation and vascularization has important prospects.

Existing studies have promoted angiogenesis by delivering exogenous growth factors, such as vascular endothelial growth factor (VEGF) and basic fibroblast growth factor (bFGF). However, a key limitation is that a single factor often cannot achieve the spatiotemporal coordinated regulation of multiple signals that occurs in natural repair processes. For example, in bone repair, angiogenesis must be coupled with osteoblast differentiation, while in skin healing, neovascularization must be synchronized with epithelial reconstruction and collagen remodeling. In recent years, biomaterial design has gradually considered integrating immune modulation and angiogenic signals to mimic the dynamic feedback mechanisms of the natural microenvironment. GM-CSF, as a multifunctional cytokine, demonstrates the advantage of integrating multiple functions. As an important regulator of myeloid cell development and activation, it is a key molecule connecting innate immunity and tissue repair [6] and participates in tissue injury repair with temporal and spatial specificity. In the early stages of injury, GM-CSF rapidly recruits neutrophils and monocytes to the damaged site. It promotes the polarization of macrophages toward a pro-inflammatory (M1) phenotype [7]. As repair progresses, it regulates the transition of macrophages toward a reparative (M2) phenotype and stimulates their secretion of factors such as VEGF and transforming growth factor-beta TGF-β [8, 9], thereby providing a microenvironment for angiogenesis and matrix remodeling. In addition, GM-CSF can directly bind to receptors on vascular endothelial cells, promoting the proliferation, migration and tube formation of Human umbilical vein endothelial cells (HUVECs) [10, 11]. It can also regulate the proliferation, migration and differentiation of cells in tissues such as epithelial cells and osteoblasts. Therefore, GM-CSF plays a central role in various repair processes, including skin wound healing and bone regeneration [10–12]. However, despite its considerable potential in various tissue repair processes, the clinical application of GM-CSF still has limitations. GM-CSF has an *in vivo* half-life of only a few hours and is prone to rapid diffusion and hydrolysis in physiological fluids [13]. It has a strong ability to recruit cells, but without anchoring, this leads to recruited cells failing to effectively settle and function at the site of injury. This mismatch between cytokine delivery and localization has become a common issue limiting the clinical translation of many cytokine-based therapies.

Fusion proteins represent a potent strategy in this context, exemplified by the fibroblast growth factor 21-elastin-like polypeptide (FGF21-ELP) fusion, which enhances stability and provides sustained release to coordinate inflammation modulation, collagen synthesis and angiogenesis for diabetic wound healing and the platelet-derived growth factor BB-laminin LG4 domain (PDGFB-LG4) fusion, which achieves localized, heparin-mediated sustained delivery to synergistically promote vascularization and osteogenesis in bone defects [14, 15]. Previous studies have employed fusion protein strategies, linking GM-CSF to human serum albumin, to prolong its circulation half-life [13]. Furthermore, fusion of GM-CSF with a tumor-targeting moiety, such as the anti-GD2 chimeric antibody Ch14.18, can specifically deliver it to the tumor microenvironment, thereby potently activating dendritic cells and exerting stronger antitumor immunity [16]. While these strategies improve systemic exposure or specific targeting, they do not inherently provide a mechanism for the recruited cells to stably adhere and engage with the injury site matrix. Therefore, finding a molecule that can effectively immobilize GM-CSF at the wound site while providing adhesion signals for recruited cells has become key to overcoming this problem. Notably, vitronectin (VTN), which is highly expressed in the eggshell membrane [17], contains Arg-Gly-Asp (RGD) sequence that can anchor cells and has become an effective candidate molecule to assist GM-CSF in its function. The RGD sequence in VTN can also efficiently mediate cell adhesion and proliferation (e.g. endothelial cells) through integrins αvβ3/αvβ5 [18, 19]. In addition, VTN can activate the PI3K-Akt pathway to inhibit cell apoptosis, sustaining the survival, self-renewal ability and clonogenic capacity of stem cells (e.g. mesenchymal stem cells) under stress conditions like serum deprivation [20]. Studies have shown that VTN also participates in regulating fibrinolysis and wound contraction [21, 22] and can influence the inflammatory microenvironment and angiogenesis process through interactions with the Mac-1 receptor [23].

Based on the functionality of VTN, it can effectively compensate for GM-CSF’s inability to anchor cells during recruitment, while also synergistically enhancing the regulatory effects of GM-CSF. Therefore, this study utilized genetic recombination technology to construct a fusion protein by linking the gene sequences of VTN and GM-CSF and expressing the fusion protein (VTN-GM-CSF, VGC). The core innovation of this design lies in leveraging the strong chemotactic signal of GM-CSF for efficient cell recruitment, while using the RGD sequence of VTN and its specific binding ability to cell surface integrins αvβ3 and αvβ5 to provide stable anchoring.

**Figure 1:**
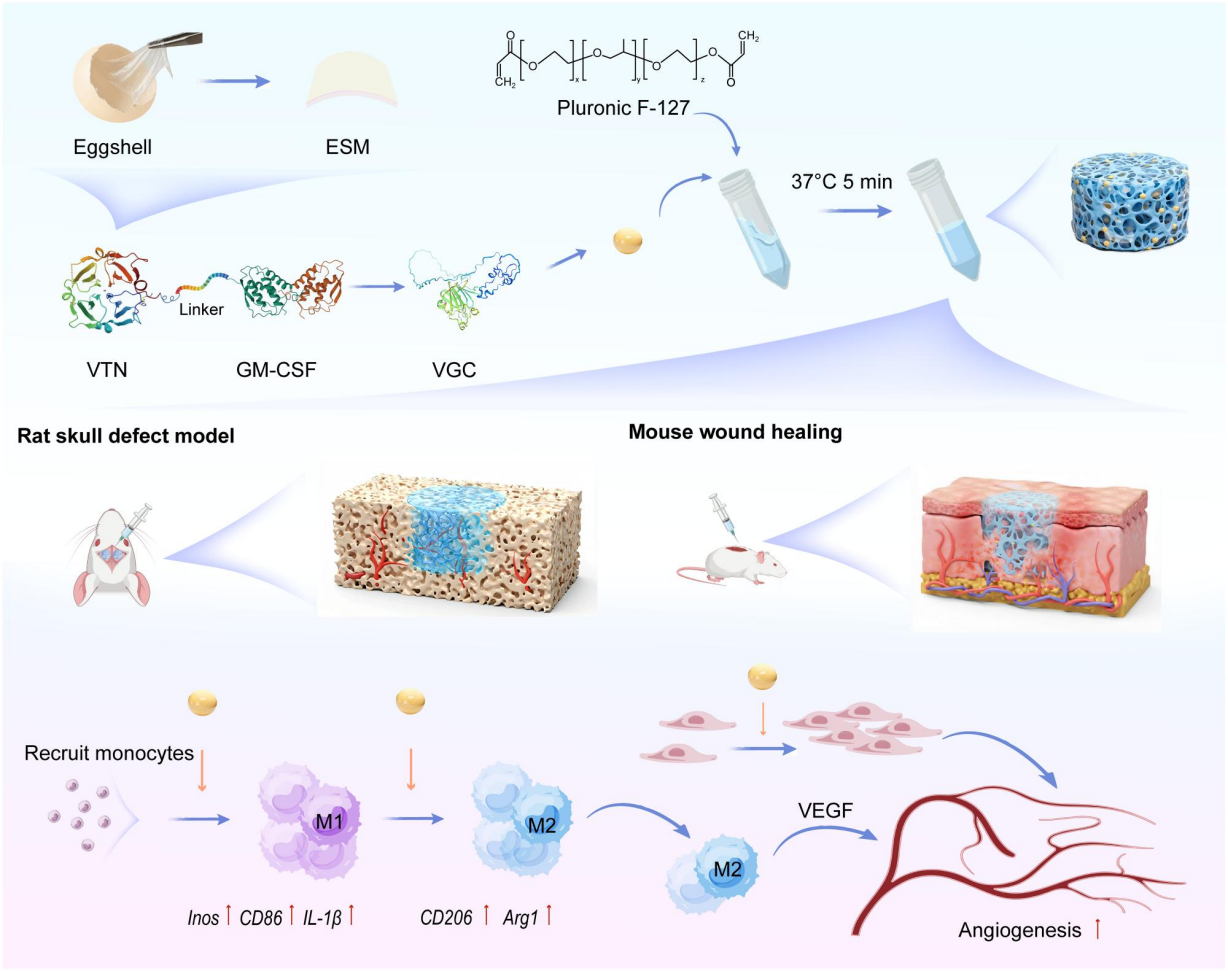
Construction and functional exploration of a new type of bionic fusion protein hydrogel. Inspired by the tissue repair function of natural eggshell membranes, we genetically fused VTN, which was a highly expressed protein in these membranes, with the immunomodulatory cytokine GM-CSF to create a fusion protein, VGC. Then VGC was incorporated into thermosensitive pluronic F-127 to form an injectable, three-dimensional porous hydrogel (F-VGC) at body temperature (37°C). F-VGC significantly enhanced bone and skin tissue repair. Mechanistically, it effectively recruited monocytes to the injury site and promoted their polarization from a pro-inflammatory M1 state (characterized by high *CD86* and *IL-1β*) to a pro-reparative M2 state (with high *CD206* and *Arg1*), thereby shifting the immune microenvironment from inflammatory to regenerative. Furthermore, VGC directly stimulated the proliferation, migration and survival of endothelial cells, while the activated M2 macrophages upregulated VEGF expression. This synergy drove angiogenesis, establishing the foundation for the *in situ* regeneration of both bone and skin tissues. Created with BioGDP.com [24].

This establishes a closed-loop repair mechanism of ‘recruitment-anchoring-regulation’. Furthermore, as the carrier material degrades, the release rate of GM-CSF precisely aligns with the tissue regeneration timeline. This approach avoids the excessive cell activation caused by sustained cytokine stimulation, while utilizing the material-mediated microenvironmental regulation to achieve *in situ* tissue repair and functional reconstruction at the injury site. To broaden the application of this protein material, this study further incorporated VGC fusion proteins into thermosensitive F127 hydrogels. Leveraging the injectability and sustained-release properties, the comprehensive efficacy of VGC in coordinating inflammatory chronology, promoting vascularization and accelerating functional tissue reconstruction was systematically evaluated in two representative animal models: a rat calvarial defect model (representing slow hard tissue repair) and a mouse full-thickness skin defect model (representing rapid soft tissue regeneration), with the aim of providing new insights for developing *in situ* regeneration-inducing materials (Figure 1).

## Materials and methods

### Design and construction of the VGC fusion protein

The VGC fusion protein was constructed by fusing the functional domains of human VTN and GM-CSF with a (GGGGSGGGGS) flexible linker peptide (Tables S2) and was designed to include a His-tag. The flexible linker was incorporated to confer conformational freedom and steric flexibility between the VTN and GM-CSF moieties, thereby preventing functional interference. The gene was then cloned into a pET-28a vector using the *Nde I* and *Hind III* restriction sites to generate the recombinant plasmid pET28a-VGC (Figure S1), which was subsequently transformed into *E. coli* BL21(DE3) for expression. Following validation by SDS-PAGE and Western blot (WB), the target protein was purified, analyzed by mass spectrometry, lyophilized and stored at −20°C.

### Structural analysis of the VGC fusion protein

The three-dimensional structure of the VGC fusion protein was predicted using the Swiss-Model server. The secondary structure of the purified protein was analyzed by circular dichroism (CD) spectroscopy.

### Preparation and characterization of VGC hydrogel

A 30% (w/v) thermosensitive hydrogel was prepared by dissolving Pluronic F127 in phosphate-buffered saline (PBS) (Figure S2). F-VTN and F-VGC hydrogels were subsequently fabricated by incorporating VTN or VGC protein, respectively. The surface wettability of air-dried gels was measured using the static contact angle method. Mechanical properties were characterized with a rheometer, and the internal microstructure was examined by scanning electron microscopy (SEM). The swelling behavior was assessed by means of immersing pre-weighed hydrogel samples in PBS at 37°C, with periodic weighing to record mass changes. The swelling ratio was calculated using Equation (1):


(1) Swelling ratio (%)=ws-wdwd×100%

*W_s_* is the initial weight, and *W_d_* is the weight at each time point.

### Distribution and release of VGC fusion protein in hydrogels

Protein loading in the hydrogels was verified by immunofluorescence staining and visualized using confocal microscopy. To characterize the release kinetics, F-VTN and F-VGC hydrogels were incubated in PBS at 37°C with gentle agitation. The supernatant was collected at predetermined time points, and the protein concentration in the release medium at the *n*^th^ sampling point (*C_n_*) was quantified using a micro‑BCA assay. The cumulative amount of protein released by the *n*^th^ time point (*Q_n_*) was calculated using Equation (2):


(2) Qn=CnV0+V∑i=1n−1Ci

*V_0_* is the initial volume and *V* is the sample volume withdrawn at each interval.

The cumulative release percentage was then calculated using Equation (3):


(3) Cumulative release (%)=QnM0×100%

*M_0_* is the initial total protein loaded into the hydrogel.

### Effects of VGC fusion protein on angiogenesis

#### HUVEC culture

HUVECs were cultured in Dulbecco’s Modified Eagle Medium (DMEM) with 10% fetal bovine serum (FBS) and 1% penicillin-streptomycin at 37°C in a 5% CO_2_ incubator.

#### Cell proliferation

HUVECs (2 × 10^4^ cells/mL) were seeded in 96-well plates and treated with 20 µg/mL of VTN or VGC, a concentration optimized in preliminary experiments (Figure S3A), with basal medium as the control. Cell proliferation was assessed on Days 1, 2, 3 and 5 using CCK-8. In parallel, cell morphology was examined by staining F-actin with phalloidin and imaging with confocal microscopy.

#### Cell migration

HUVECs (2 × 10^5^ cells/mL) were seeded in 12-well plates and allowed to adhere and grow to confluence. Subsequently, a scratch wound was created. Cells were then treated with 20 µg/mL VTN or VGC in basal medium, and basal medium alone served as the control. Following 24 and 48 h of treatment, the cells were fixed with 4% paraformaldehyde, stained with 0.1% crystal violet and imaged. Cell migration was quantified by ImageJ software.

#### Tube formation assay of HUVECs

Pre-Matrigel was coated onto 96-well plates and allowed to polymerize at 37°C. HUVECs (3 × 10^6^ cells/mL) were then seeded on the Matrigel and treated with VTN or VGC at a final concentration of 100 μg/mL for 8 h (a concentration optimized in preliminary experiments; Figure S3B). Tube formation was observed with an inverted fluorescence microscope. The acquired images were analyzed using ImageJ software.

#### Chick embryo chorioallantoic membrane (CAM) assay

Fertilized chicken eggs were incubated for 3–7 days. A small window was opened in the eggshell above the CAM, and 100 μg/mL solutions of VTN or VGC were applied onto the membrane, with PBS as a control. The window was then resealed, and the embryos were incubated for an additional 72 h. Angiogenesis on the CAM was observed with a stereomicroscope and quantified using ImageJ software.

### The role of VGC protein in macrophage chemotaxis and phenotypic regulation

#### Macrophage culture

RAW264.7 macrophages were cultured in DMEM with 10% FBS and 1% penicillin-streptomycin at 37°C in a 5% CO_2_ incubator.

#### Transwell migration assay

RAW264.7 macrophages were seeded in the upper chamber of a Transwell insert (8 μm pore) at a density of 5 × 10^4^ cells per well. Basal medium containing 20 μg/mL VTN or VGC was placed in the lower chamber. After 10 h of culture, cells that had migrated to the lower side of the membrane were fixed with 4% paraformaldehyde and stained with 0.1% crystal violet. Images were captured using an inverted microscope, and the number of migrated cells was quantified.

#### Analysis of macrophage phenotype-related gene expression

To establish an inflammatory model, RAW264.7 macrophages were first stimulated with 100 ng/mL lipopolysaccharide (LPS) for 3 h. Cells were then treated with 20 μg/mL of VTN or VGC. Total RNA was extracted using Trizol and reverse-transcribed into cDNA. With *GAPDH* as an internal control, quantitative polymerase chain reaction (qPCR) was performed to measure the mRNA levels of M1 markers (*Inos, CD86, IL-1β*) at 6 h post-treatment to assess the early inflammatory response, and M2 markers (*Arg1*, *CD206*) at 48 h to evaluate phenotypic modulation. Relative gene expression was calculated and statistical analysis was performed.

#### WB analysis of M2 macrophage markers

Following the aforementioned treatment, total protein was extracted at 48 h. The expression of the M2 markers Arg1 and CD206 was analyzed by WB, using GAPDH as a loading control. Protein band intensity was quantified with ImageJ software.

### Cell compatibility and chemotactic effect of the VGC protein

The effects of VGC on cell proliferation and migration were assessed using human dermal fibroblasts (HDFs; Wuhan Shang’en Biotechnology) and mouse embryonic osteoblast cells (MC3T3-E1; Shanghai Saibai Kang) following the protocols described in Cell proliferation and Cell migration sections, respectively. The cell recruitment capacity of VGC was evaluated using primary bone marrow-derived mesenchymal stem cells (BMSCs) as described in Transwell migration assay section.

### *In vivo* evaluation of VGC hydrogel in tissue repair

Experimental animals were obtained from the Experimental Animal Centre of Xi’an Jiaotong University School of Medicine, with animal experiment license number: SCXK (Shaanxi) 2018-001. All animal experimental procedures were approved by the Animal Ethics Review Committee of Northwest University (IACUC, ACUC2013015).

#### Rat cranial defect repair model

Bilateral, critical-size calvarial defects (5 mm in diameter) were created in 8-week-old male Sprague-Dawley (SD) rats. Animals were randomly divided into four groups (*n* = 3): blank control group, F127, F-VTN and F-VGC hydrogel groups. Cranial samples were collected at 8 and 12 weeks post-surgery and fixed in 4% paraformaldehyde. The defect sites were scanned by micro-computed tomography (Micro-CT). Bone volume fraction (BV/TV), trabecular thickness (Tb.Th), trabecular separation (Tb.Sp) and bone mineral density (BMD) were calculated. The samples were then processed for morphology, histology and immunofluorescence evaluation.

#### Murine full-thickness skin wound healing model

Eight-week-old male Kunming mice were used to establish full-thickness skin defect models. Wound healing was monitored by digital photography, and the wound closure rate was calculated. Skin tissue specimens were collected on postoperative Days 3, 7, 14 and 21. The samples were fixed in 4% paraformaldehyde, sectioned for histological and immunofluorescence analysis.

#### Histological and immunofluorescence analysis

Bone specimens were decalcified in 10% EDTA for 8 weeks, while skin tissues required no decalcification. Subsequently, all samples were embedded, sectioned at 4 μm thickness and stained with hematoxylin and eosin (H&E) or Masson’s trichrome (MTC) for microscopic examination. For immunofluorescence, sections were dewaxed and then subjected to antigen retrieval. After blocking with 5% Bovine Serum Albumin (BSA), sections were incubated with primary and secondary antibodies. Nuclei were counterstained with 4',6-Diamidino-2-Phenylindole (DAPI), and images were acquired using a laser scanning confocal microscope. Detailed antibody information is provided in Table S1.

### Statistical analysis

The data were analyzed using GraphPad Prism 10.0 statistical software and presented as mean ± standard deviation (SD). An unpaired student’s *t*-test was used when comparing two groups. For comparisons between three or more groups, one-way analysis of variance (ANOVA) with post-hoc Tukey HSD test was used. Statistically significant differences were set at *P* < 0.05.

## Results and discussion

### Fusion protein expression and purification

**Figure 2:**
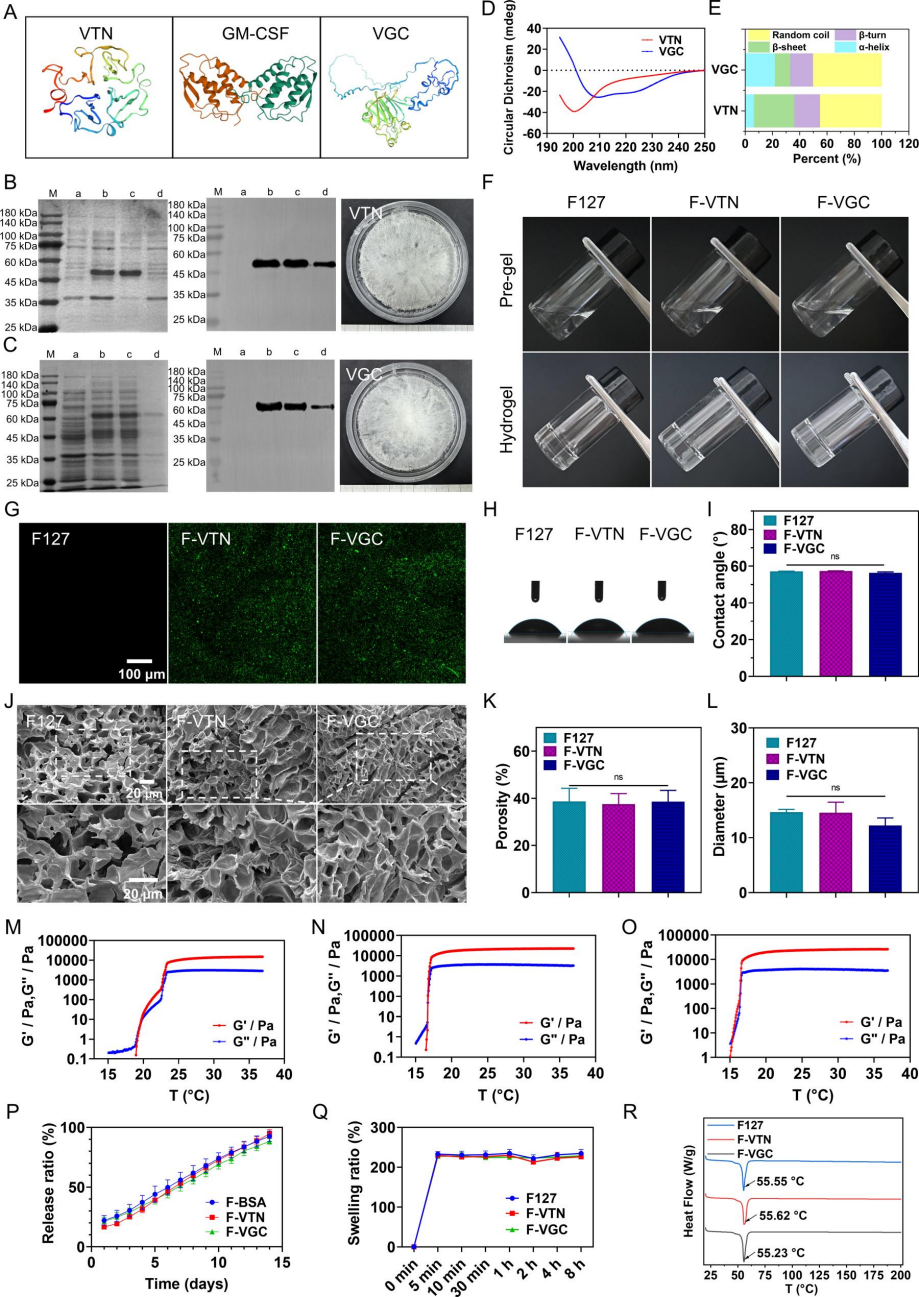
Purification of VGC protein and characterization of VGC hydrogel. (**A**) Predicted tertiary structures of VTN, GM-CSF and VGC proteins. The VTN and GM‑CSF domains are linked by a flexible linker and remain spatially independent, preserving their respective folds. SDS-PAGE, WB (anti-VTN antibody) and images of lyophilized proteins for (**B**) VTN and (**C**) VGC. Lane designations: M, marker; a, uninduced crude extract; b, induced crude extract; c, supernatant after ultrasonication; d, pellet after ultrasonication. (**D**) CD spectra of VTN and VGC, with (**E**) Statistical distribution of secondary structure (α-helix, β-sheet, β-turn and random coil fractions). CD confirms that the characteristic structural elements of VTN and GM‑CSF are retained in the VGC fusion. (**F**) Morphology of F127, F-VTN and F-VGC before and after gelation show that protein‑loaded gels exhibit clarity similar to that of pure F127. (**G**) Distribution of fluorescently labeled proteins (green) within the hydrogels, with no signal in the PBS control. (**H**) Water contact angle measurements and (**I**) corresponding statistical analysis. Protein loading does not significantly alter surface wettability. (**J**) Representative SEM images of hydrogels. (**K**) Porosity and (**L**) Pore size of hydrogels. All formulations display a honeycomb‑like, interconnected porous network. Frequency sweep tests showing the elastic modulus (G′) and viscous modulus (G″) for (**M**) F-127, (**N**) F-VTN and (**O**) F-VGC hydrogels. All undergo sol–gel transition (G′ > G″) below ∼20 °C and reach G′ ∼10^4^ Pa. (**P**) Cumulative release profile of proteins from the hydrogel are similar, demonstrating sustained release during 15 days. (**Q**) Swelling kinetics of hydrogels. (**R**) DSC thermograms of hydrogels. All groups exhibit sharp endothermic melting peaks with no significant shifts. All data are presented as mean ± standard deviation.

The predicted structure of our constructed VGC fusion protein was shown in Figure 2A. The VTN and GM-CSF domains, connected by a flexible linker, remained spatially independent to preserve their respective structural integrity and function [25]. The expressed VTN (Figure 2B) and VGC fusion protein (Figure 2C) exhibited apparent molecular weights consistent with their theoretical values (∼50 kDa and ∼62 kDa, respectively). WB analysis confirmed the identity of these bands as VTN and VGC (Figure 2B and C). SDS-PAGE analysis for the purified VTN protein showed a single, clear band with a purity of 97.5% (Figure S1D) and the purified VGC fusion protein showed a major band with 95.2% purity (Figure S1E), which was further analyzed by mass spectrometry (Figure S1G). Both proteins were subsequently lyophilized (Figure 2B and C). Analysis of the predicted tertiary structures (Figure 2A) and the CD spectra (Figure 2D and E) indicated that the characteristic structure elements of both VTN and GM-CSF were maintained in the VGC fusion protein. In summary, we successfully obtained high-purity VTN and VGC proteins with confirmed identity.

### Preparation and physicochemical characterization of VGC hydrogels

The temperature-sensitive F127 hydrogel enabled rapid gelation upon injection while maintaining good biocompatibility and preserving the activity of loaded proteins [26]. Before gelation occurred, all three kinds of pre-hydrogels (F127, F-VTN, F-VGC) showed a rheological liquid condition, remained fully transparency and had no precipitation, flocculation or stratification. After being incubated at 37°C for 5 minutes, each solution (F127, F-VTN, F-VGC) formed a stable, self-supporting gel (Figure 2F). Macroscopically, the protein-loaded gels (F-VTN, F-VGC) showed clarity comparable to the pure F127 control (Figure 2F).

Immunofluorescence revealed uniform distribution of VTN and VGC within the F127 hydrogel (Figure 2G), while no fluorescence was detected in the PBS control group. Water contact angle measurements (Figure 2H and I) indicated that all hydrogel samples (F127, F-VTN and F-VGC) exhibited water contact angles below 60°, indicating hydrophilic surfaces with no significant alteration in wettability due to protein loading.

SEM showed that F127, F-VTN and F-VGC possessed a honeycomb-like porous structure with interconnected pores (Figure 2J). The average pore size was 14.67 ± 3.68 µm for F127, 14.66 ± 4.00 µm for F-VTN and 12.24 ± 2.93 µm for F-VGC (Figure S4), with corresponding porosities of 38.64 ± 5.58%, 37.59 ± 4.38% and 38.60 ± 4.73%, respectively (Figure 2K). No statistically significant differences in pore size or porosity were found among the groups (Figure 2K and L), indicating that VTN or VGC protein loading did not alter the internal microarchitecture of the F127 hydrogel.

Rheological analysis verified that all hydrogels have thermosensitive gelation property, with a sol-gel transition (G′ > G″) happening under 20°C and a storage modulus (G′) arriving at ∼10^4^ Pa, indicative of a stable elastic network (Figure 2M–O). Protein loading did not change the gelation behavior or the final mechanical strength. *In vitro* release curves of BSA, VTN and VGC from the hydrogels were similar, demonstrating sustained release during 15 days and consistent loading/release abilities for different proteins (Figure 2P).

Swelling tests showed all hydrogels reached equilibrium in PBS at 37°C within 10 minutes, with comparable swelling ratios (Figure 2Q). Differential scanning calorimetry (DSC) revealed sharp endothermic melting peaks for all groups, with no significant differences, confirming that protein incorporation did not affect the thermal stability of the F127 matrix (Figure 2R).

### Angiogenic effects of the VGC protein

**Figure 3:**
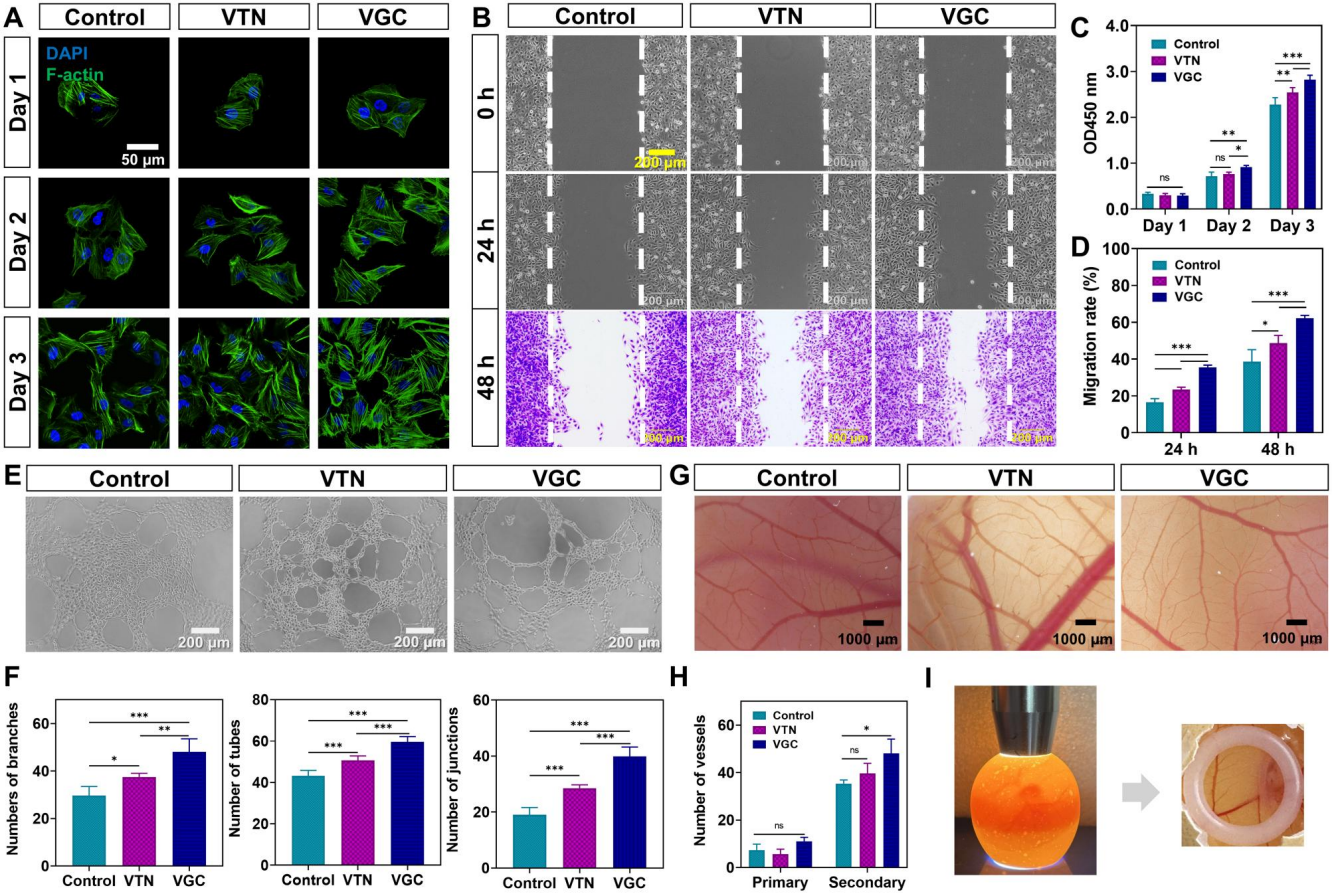
VGC Protein enhances angiogenesis. (**A**) HUVEC morphology (DAPI/phalloidin) after culture with VTN or VGC (20 μg/mL, Days 1-3). (**B**) Migration at 24, 48 h. (**C**) Proliferation (CCK-8, *n* = 6). (**D**) Migration rate quantification (*n* = 6). (**E**) Representative images of tube formation with VTN or VGC (100 μg/mL). Compared with the control and VTN groups, the VGC group significantly promoted HUVEC proliferation and migration. (**F**) Number of tubes, branches, junctions quantified (*n* = 6). Compared with the control and VTN groups, the VGC group significantly promoted tube formation of HUVEC *in vitro*. (**G**) Representative images of CAM assay with VTN or VGC (100 μg/mL). (**H**) Primary/secondary vessel counts (*n* = 3). Compared with the control group, VGC significantly promoted the formation of secondary vessels. (**I**) CAM model schematic. All data are presented as mean ± standard deviation. *P < 0.05, **P < 0.01, ***P < 0.001.

The pro-angiogenic potential of the VGC fusion protein evaluated using HUVECs. Cells treated with VTN or VGC displayed enhanced spreading and increased density over time, suggesting biocompatibility and a potential pro-proliferative effect (Figure 3A). CCK-8 assay showed that both VTN and VGC significantly promoted HUVEC proliferation compared to the control group (Control: 0.33 ± 0.04, 0.72 ± 0.09, 2.28 ± 0.14 on Days 1, 2, 3; VTN: 0.30 ± 0.03, 0.76 ± 0.04, 2.54 ± 0.11; VGC: 0.30 ± 0.04, 0.91 ± 0.03, 2.82 ± 0.10) (Figure 3C). Notably, the VGC fusion protein exhibited a stronger proliferative effect than VTN alone at all time points.

The scratch assay was used to evaluate the effects of these proteins on HUVEC migration (Figure 3B). VGC exhibited the strongest pro-migration effect at both 24 h and 48 h. Quantitative analysis (Figure 3D) showed that after 24 h, the migration rate was significantly higher in the VGC group (35.52 ± 1.17%, ***P < 0.001) than in the VTN (23.45 ± 1.24%) and control (16.57 ± 1.90%) groups. This superior effect persisted at 48 h, with migration rates of 62.30 ± 1.42% for VGC (***P < 0.001), 48.69 ± 4.15% for VTN and 38.64 ± 6.46% for the control, confirming the sustained and highly effective migration-promoting capacity of VGC.

Matrix gel tube formation assays (Figure 3E) demonstrated that, compared to VTN, VGC induced HUVECs to form more complex and dense tubular structures. Specifically, the number of branches was increased in the VTN group (37.50 ± 1.52, *P < 0.05) and the VGC group (48.00 ± 5.55, ***P < 0.001) compared to the control (29.67 ± 3.83). The total number of tubes was also significantly greater in both the VTN (50.67 ± 2.16, ***P < 0.001) and VGC (59.67 ± 2.50, ***P < 0.001) groups than in the control group (43.17 ± 2.64). Moreover, the number of tube junctions was markedly higher in the VTN (28.50 ± 1.23, ***P < 0.001) and VGC (39.83 ± 3.37, ***P < 0.001) groups compared to the control (19.00 ± 2.61). Notably, VGC demonstrated significantly higher efficacy than VTN alone in promoting HUVEC tubulation. These results suggested a positive synergistic interaction between the GM-CSF and VTN domains in promoting tube formation.

The pro-angiogenic activity of VGC was further evaluated *in vivo* using the CAM assay (Figure 3I). VGC treatment significantly promoted capillary proliferation relative to the control (Figure 3G). Quantitative analysis showed that the number of secondary vessels was significantly higher in the VGC group (48.00 ± 6.08) than in the control group (35.33 ± 1.53, *P < 0.05). The VTN group (39.67 ± 4.16) showed a slight, but statistically non-significant, increase compared to the control (*P* > 0.05, Figure 3H). These findings indicated that VGC effectively promoted the formation of complex capillary networks *in vivo*. Notably, GM-CSF is a key regulator of angiogenesis, activating endothelial proliferation and vascular remodeling signals by upregulating VEGF expression and modulating the Ang-1/Ang-2 ratio, thereby supporting tissue repair through enhanced blood supply [27]. This mechanism aligned with the observed significant promotion of endothelial cell migration, tubulogenesis and *in vivo* angiogenesis by VGC in this study.

In summary, VGC’s exceptional pro-angiogenic effects might have stemmed from its dual integration of GM-CSF’s direct stimulation of endothelial cell proliferation and migration [28], alongside VTN’s provision of endothelial cell adhesion and survival signals [29], thereby synergistically promoting the formation of complex vascular networks.

### Effects of the VGC fusion protein on macrophage chemotactic migration and phenotypic modulation

**Figure 4:**
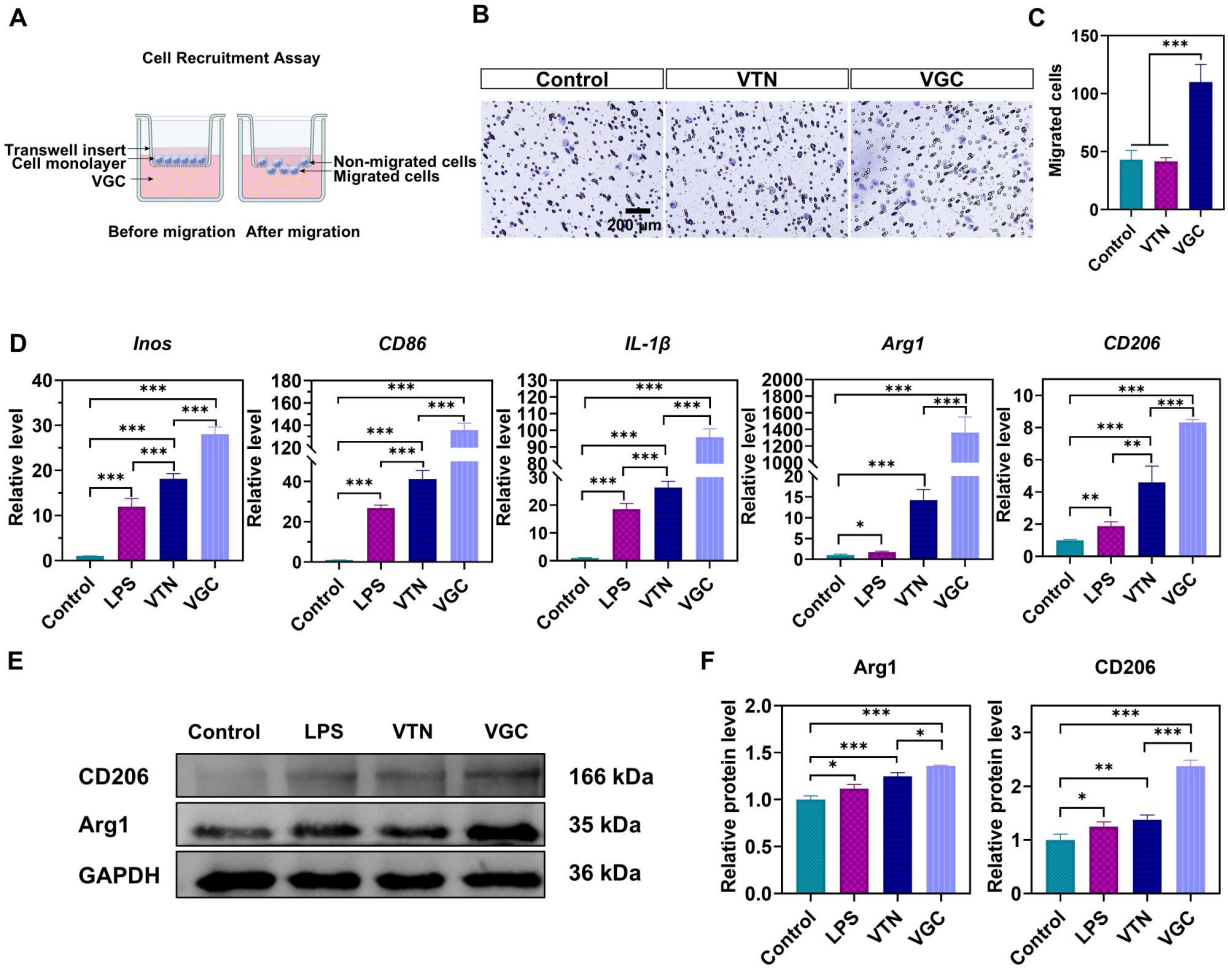
VGC fusion protein drives macrophage recruitment and M2 polarization. (**A**) Schematic illustration of the transwell migration experiment. VGC enhances the chemotactic migration of RAW264.7 macrophages over 10 h, as shown by (**B**) representative crystal violet-stained images and (**C**) quantified cell counts, *n* = 6. (**D**) At 6 h, the expression levels of *Inos*, *CD86*, and *IL-1β* (M1) in macrophages are significantly higher in the VGC group than in both the LPS-induced group and the VTN group (*P* < 0.001). At 48 h, the expression levels of *Arg1* and *CD206* (M2) in macrophages are significantly higher in the VGC group than in both the LPS-induced group and the VTN group (*P* < 0.001). VGC promotes an M2-polarized phenotype, evidenced by the mRNA expression profile of polarization markers. (**E**) Representative WB bands for Arg1 and CD206 after 48-h treatment and (**F**) their corresponding densitometric analysis, *n* = 3. All data are presented as mean ± standard deviation. *P < 0.05, **P < 0.01, ***P < 0.001.

The chemotactic effect of VGC on macrophages was evaluated using a Transwell assay (Figure 4A and B). Quantitative analysis (Figure 4C) showed that, compared to the control, VTN did not significantly promote RAW264.7 macrophages migration (*P* > 0.05). In contrast, VGC significantly enhanced cell migration (110.0 ± 15.23 cells; ***P < 0.001), indicating that the fusion protein exhibited enhanced potency in regulating macrophage chemotaxis.

Macrophage plasticity and polarization were central to immunoregulation and tissue repair processes. M1 macrophages highly expressed pro-inflammatory factors such as inducible nitric oxide synthase (*Inos*), *CD86* and pro-inflammatory factors such as interleukin-1β (*IL-1β*). In contrast, M2 macrophages highly expressed anti-inflammatory and repair-related molecules including *Arg1* and *CD206*. To investigate the effects of the VGC fusion protein on macrophage polarization, RAW264.7 macrophages were first treated with LPS for 3 h to prime an inflammatory state. Cells were then incubated with 20 μg/mL VTN or VGC and changes in M1/M2 marker expression were assessed.

qRT-PCR analysis revealed distinct temporal effects on macrophage polarization markers (Figure 4D). After 6 h of co-culture, LPS significantly upregulated the expression of M1 markers *Inos*, *CD86* and *IL-1β* compared to the control (*Inos*: 11.94 ± 1.83, *CD86*: 26.82 ± 1.48, *IL-1β:* 18.57 ± 2.05, ***P < 0.001). This induction was further enhanced by VTN and was most potent with VGC (*Inos*: 28.01 ± 1.63, *CD86*: 135.6 ± 6.39, *IL-1β:* 95.92 ± 5.04, ***P < 0.001), demonstrating that VGC most strongly promoted early M1 polarization. Conversely, after 48 h, LPS only slightly increased the expression of the M2 markers *Arg1* (1.72 ± 0.24, *P < 0.05) and *CD206* (1.88 ± 0.26, **P < 0.01). Both VTN and VGC significantly induced high expression of these markers, with VGC again showing the strongest effect (*Arg1*: 1362 ± 188.9; *CD206*: 8.31 ± 0.19; ***P < 0.001 for both).

WB analysis of the 48 h samples corroborated these findings at the protein level, revealing differential expression of Arg1 and CD206 (Figure 4E). VGC significantly upregulated the protein levels of both CD206 (1.34 ± 0.02, ***P < 0.001) and Arg1 (2.37 ± 0.06, ***P < 0.001) compared to the control. VTN also significantly promoted the expression of Arg1 (1.25 ± 0.02, ***P < 0.001) and CD206 (1.37 ± 0.05, **P < 0.01), although its effect on Arg1 was less pronounced than that of VGC (Figure 4F).

Macrophage phenotypic polarization was a pivotal regulator of scaffold vascularization, where the early M1 phenotype initiated vascular sprouting and the subsequent shift to the M2 phenotype promoted the maturation of stable vasculature via the secretion of factors like VEGF and PDGF [30]. This study demonstrated that VGC materials precisely guided these processes by directly activating endothelial cells, promoting their proliferation and initiating vascular remodeling signals (Figure 3). Based on this, we hypothesized that within complex tissue reconstruction environments, the GM-CSF component in VGC might shape a regenerative-favorable immune microenvironment by driving macrophage conversion toward the reparative M2 phenotype [7, 8, 20], thereby systemically amplifying pro-angiogenic efficacy. Furthermore, the VTN module provided essential adhesion and survival signals for endothelial cells through its RGD sequence [28, 29], a mechanism aligned with studies showing that VTN -functionalized surfaces accelerated vascular regeneration [31].

Functionally, both VTN and VGC enhanced the collective, two-dimensional migration of HUVECs (Figure 3B and D), an effect primarily attributable to the pro-adhesive microenvironment furnished by the VTN domain. In contrast, Transwell/cell recruitment assays revealed that VGC, but not VTN alone, effectively stimulated the directed migration of RAW264.7 macrophages (Figure 4A–C). This directly evidences that the GM-CSF component is responsible for mediating the homing and recruitment of reparative cells. Thus, VGC established a temporally coordinated system: GM-CSF recruited and modulated immune cells to optimize the microenvironment for angiogenesis, while VTN provided the critical matrix anchoring and survival support for endothelial cells within that niche. This synergistic, sequential action provided a core design principle for next-generation biomaterials capable of actively steering *in vivo* repair processes.

### Cytocompatibility and functional *in vitro* assessment of the VGC fusion protein

**Figure 5:**
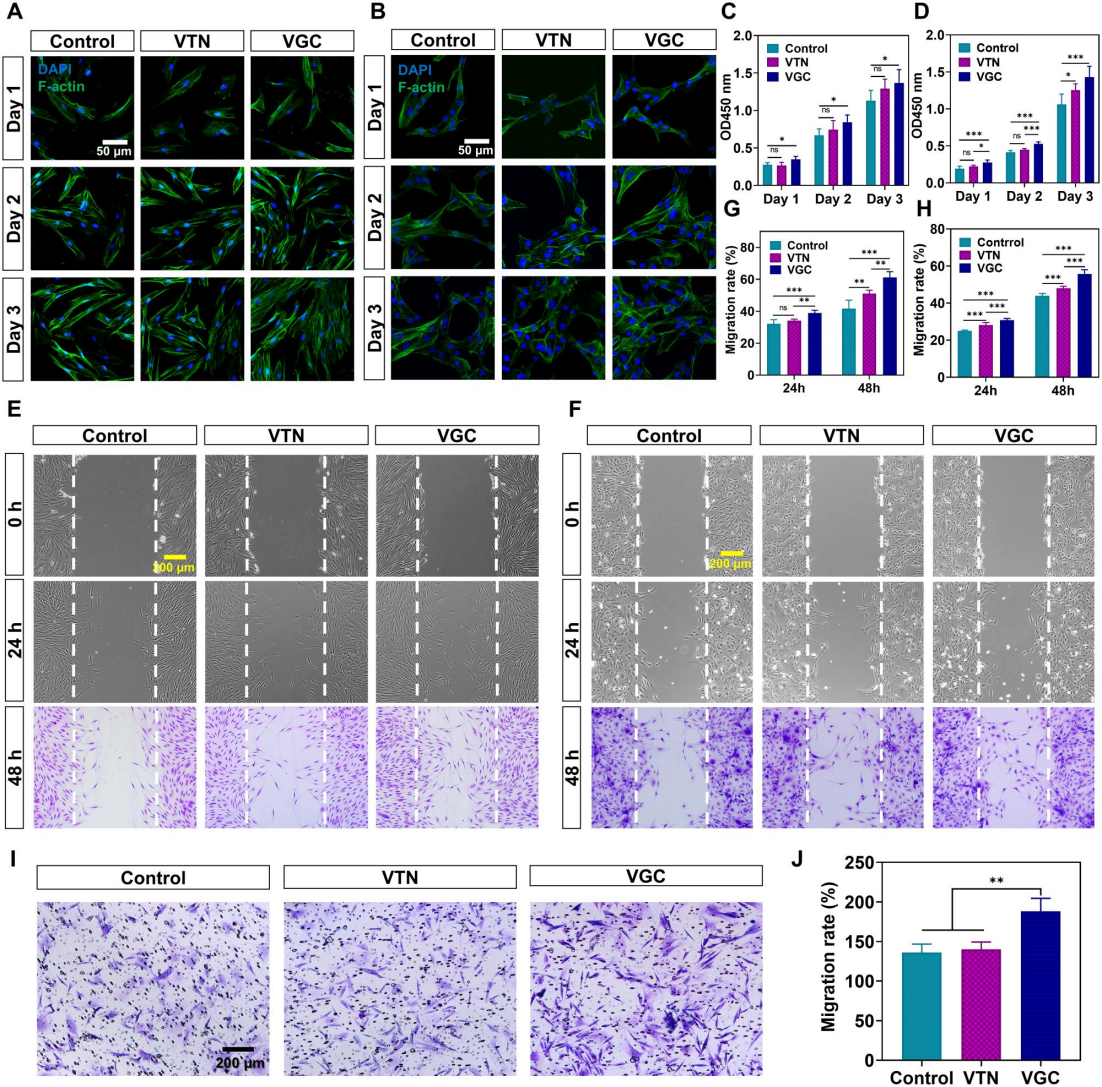
Cytocompatibility and chemotactic activity of the VGC protein. Fluorescence images of (**A**) HDFs and (**B**) MC3T3-E1 cells cultured with VTN or VGC (20 μg/mL) for 1-3 days (nuclei: DAPI/blue; cytoskeleton: phalloidin/green). Proliferation of (**C**) HDFs and (D) MC3T3-E1 cells assessed by CCK-8 assay. Compared with the control group, VGC promoted the proliferation of HDFs and MC3T3-E1. Representative images of (**E**) HDF and (**F**) MC3T3-E1 cell migration after 24 and 48 h. Quantified migration rates for (**G**) HDFs and (**H**) MC3T3-E1 cells. Compared with the Control and VTN groups, the VGC group significantly promoted the migration of HDFs and MC3T3-E1. (**I**) Chemotactic migration of BMSCs toward VTN or VGC (20 μg/mL, 10 h; crystal violet stain). (**J**) Quantification of migrated BMSCs. Compared with the Control and VTN groups, VGC exhibited a significant chemotactic effect on BMSCs (*P* < 0.01). Data are presented as mean ± standard deviation (*n* = 6); *P < 0.05, **P < 0.01, ***P < 0.001.

To further evaluate the broad effects of VGC on other key cells involved in tissue repair, we also examined its biological activity on HDFs and MC3T3-E1 cells. The results showed that VGC promoted proliferation and migration of both cells, displaying better effectiveness in comparison with the single VTN protein (Figure 5A–H). Meanwhile, VGC exhibited potent chemotactic activity toward BMSCs (Figure 5I and J) and promoted their osteogenic differentiation (Figure S5). In summary, VGC systematically built a cellular basis helpful for tissue regeneration by cooperatively controlling multiple repair-related cell types, which included immune cells, vascular endothelial cells, fibroblasts and osteoprogenitor cells. These coordinated cellular effects provided a robust cellular basis for achieving efficient repair in subsequent bone and skin defect models.

### Bone repair efficacy of VGC in a rat cranial defect model

**Figure 6:**
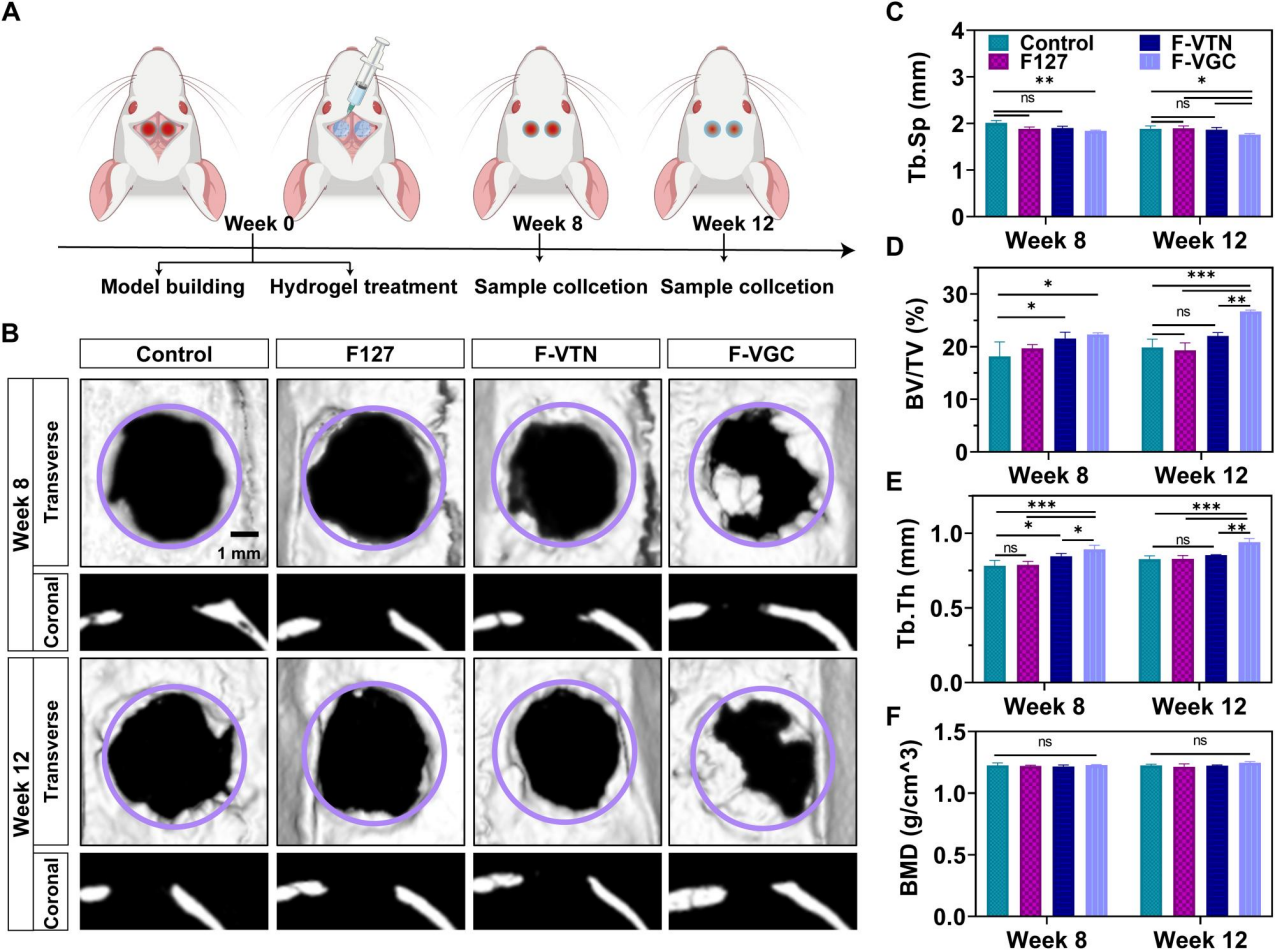
Micro-CT evaluation and bone morphometric analysis of cranial defect repair. (**A**) Schematic of the rat critical-sized calvarial defect model. (**B**) Representative cross-sectional and coronal view Micro-CT images of the defect region at 8 and 12 weeks post-operation. Quantitative statistical analysis confirms that F-VGC consistently improves bone microarchitecture, specifically reducing (**C**) Tb.Sp while increasing (**D**) BV/TV and (**E**) Tb.Th compared to controls, while (**F**). BMD remains unchanged. Data are presented as mean ± standard deviation (*n* = 3); *P < 0.05, **P < 0.01, ***P < 0.001.

Building upon the demonstrated pro-angiogenic and immunomodulatory potential of VGC, we evaluated the osteogenic efficacy of F-VGC hydrogel in a rat critical-size calvarial defect model (Figure 6A). Micro-CT analysis showed that by Week 8, newly formed bone in the F-VGC group had extended from the defect margins toward the center. By Week 12, defect closure in the F-VGC group was substantially greater than in the blank control, F127 and F-VTN groups (Figure 6B).

Quantitative micro-CT analysis revealed that F-VGC significantly improved bone microarchitecture at both time points. At 8 weeks, the F-VGC group exhibited significantly lower Tb.Sp (1.84 ± 0.02) than the control group (2.00 ± 0.06; **P < 0.01; Figure 6C). This superiority persisted at 12 weeks, with Tb.Sp in the F-VGC group (1.76 ± 0.02) being significantly lower than in all other groups (control: 1.88 ± 0.06; F127: 1.89 ± 0.06; F-VTN: 1.86 ± 0.05; *P < 0.05 for each; Figure 6C).

Similarly, BV/TV was significantly higher in the F-VGC group (22.29 ± 0.33%) than in the control group (18.17 ± 2.75%; *P < 0.05) at 8 weeks, though not significantly different from the F127 and F-VTN groups (Figure 6D). By Week 12, BV/TV in the F-VGC group (26.65 ± 0.31%) was significantly higher than in the control (***P < 0.001), F127 (***P < 0.001) and F-VTN (**P < 0.01) groups (Figure 6D).

Trabecular bone thickness (Tb.Th) was also significantly greater in the F-VGC group at both 8 weeks (0.89 ± 0.03 vs control: 0.78 ± 0.04, ***P < 0.001; F127: 0.79 ± 0.02, ***P < 0.001; F-VTN: 0.85 ± 0.02, *P < 0.05) and 12 weeks (0.94 ± 0.02 vs control: 0.83 ± 0.02, ***P < 0.001; F127: 0.83 ± 0.02, ***P < 0.001; F-VTN: 0.85 ± 0.01, **P < 0.01) (Figure 6E). BMD did not differ significantly among groups at any time point (Figure 6F).

Throughout the observation period (8–12 weeks), the F-VGC group consistently demonstrated better bone repair outcomes compared to all other groups, indicating that the F-VGC hydrogel effectively promoted cranial defect regeneration. This efficacy was mechanistically supported by previous studies showing that GM-CSF directly established a cellular and matrix foundation for bone repair by upregulating osteogenic protein expression and promoting collagen synthesis and mineralization [12]. Therefore, the significant repair efficacy of F-VGC can be primarily attributed to the direct osteogenic regulatory action of its GM-CSF component on osteoprogenitor cells.

**Figure 7:**
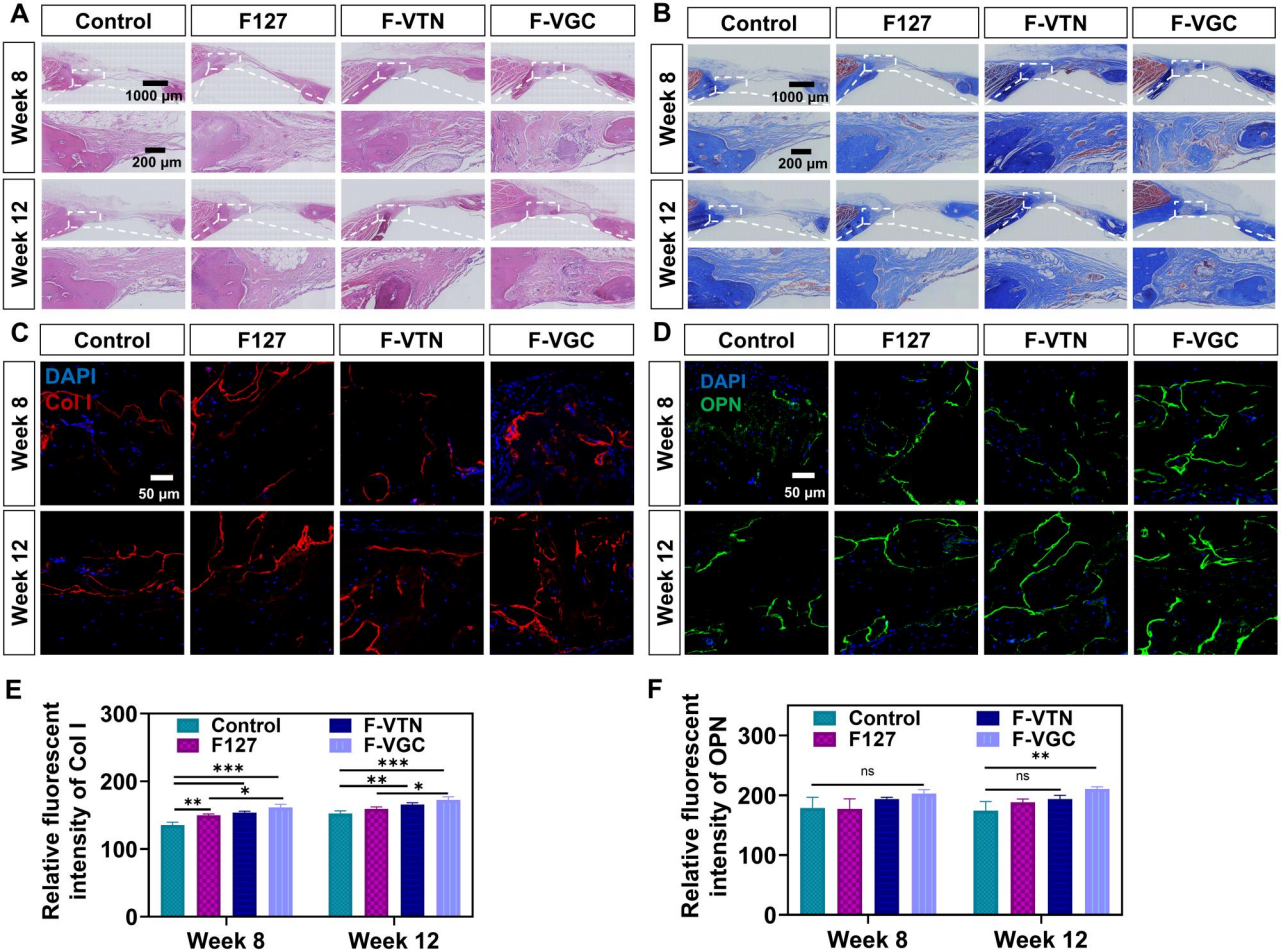
Histomorphometry and osteogenic marker analysis of bone repair. (**A**) H&E and (**B**) MTC stained sections of the calvarial defect regions at 8 and 12 weeks post-surgery. Immunofluorescence staining for (**C**) type I Col I and (**D**) OPN (scale bar: 50 μm). (**E** and **F**) Quantitative analysis of the relative fluorescence intensity for (**E**) Col I and (**F**) OPN. The deposition of Col I in the F-VGC group was significantly higher than that in the control group (*P* < 0.001) and the F127 group (*P* < 0.05). In the newly formed tissue at week 12, the level of OPN in the F-VGC group was significantly higher than that in the control group (*P* < 0.01). Data are presented as mean ± standard deviation (*n* = 3); *P < 0.05, **P < 0.01, ***P < 0.001.

Histological evaluation delineated a progressive bone regeneration process in the rat cranial defects (Figure 7). At 8 weeks, the control group showed only loose connective tissue filling the defect (Figure 7A). In contrast, the F-VTN group exhibited the formation of aligned osteoid, which by Week 12 had developed into more organized bone matrix with visible lacunae. The F-VGC group demonstrated markedly advanced repair, by Week 8, it presented compact, eosinophilic woven bone, which matured into well-defined lamellar bone containing distinct osteocyte lacunae by Week 12. MTC staining further corroborated this progressive regeneration (Figure 7B). The control group exhibited minimal collagen deposition, while the F127 group showed only sparse deposition. The F-VTN group displayed nascent bone collagen and early trabeculae. In contrast, the F-VGC group presented extensive, island-like new bone with abundant collagen deposition at the defect center as early as Week 8. By Week 12, this had developed into the most continuous, dense and connected osseous structure among all groups.

Immunofluorescence staining for collagen I (Col I) showed a time-dependent increase in collagen deposition across all groups (Figure 7C). Notably, by Week 12, the F-VGC group showed the strongest signal, which covered most parts of the defect area. At 8 weeks, Col I intensity in the F-VGC and F-VTN groups was significantly higher than that in the control group (***P < 0.001), and the F127 group also had an increase (**P < 0.01, Figure 7E). By Week 12, Col I levels in the F-VGC (***P < 0.001) and F-VTN (**P < 0.01) groups still stayed high compared with the control group, whereas the F127 group had no significant difference (Figure 7E). Therefore, the continuously high Col I expression indicated that bone matrix synthesis and mineralization were in active status. These results demonstrated that the F-VGC hydrogel effectively promoted Col I secretion and organized deposition, accelerated bone matrix remodeling and provided robust mechanical and biological support for defect repair.

Osteopontin (OPN), a critical marker of osteogenic differentiation, indicated active osteoblast maturation and bone matrix formation. In the rat cranial defect model, OPN expression was detected within the newly formed bone across all groups (Figure 7D). By Week 12, the F-VGC group exhibited stronger OPN signal intensity and a broader spatial distribution compared to the other groups (Figure 7D). Quantitative analysis of fluorescence intensity (Figure 7F) revealed comparably low OPN levels in all groups at Week 8. However, by Week 12, the relative fluorescence intensity in the F-VGC group (210.6 ± 3.6) was significantly higher than that in the control group (174.1 ± 15.4; **P < 0.01).

To assess whether this osteogenic process involved specific immune cell infiltration, we performed immunohistochemical staining for the macrophage markers CD86 (M1) and CD206 (M2) within the bone defect. Neither M1 nor M2 macrophages were detected in the newly formed tissue (Figure S6). The absence of detectable macrophage signals is likely due to the late sampling time points (8 and 12 weeks post-surgery), by which time the acute inflammatory and early reparative cell infiltration had likely resolved.

These findings suggested that F-VGC effectively promoted functional osteogenic activity *in vivo*. This process likely involved not only the pro-angiogenic and immunomodulatory potential demonstrated at the cellular level in this study (Figures 3 and 4), but also the chemotactic recruitment and regulated differentiation of key repair cells such as BMSCs. This inference was corroborated by BMSC chemotaxis experiments (Figure 5I) and aligned with prior research. For instance, GM-CSF had been shown to induce a hypoxic and proteolytic state in the bone marrow niche via C-X-C Chemokine Receptor Type 4 (CXCR4)-mediated mechanisms, thereby upregulating CXCR4 expression on BMSC and upregulating their chemotactic response to stromal cell-derived factor-1 (SDF-1) [32]. This cascade significantly increased peripheral BMSC mobilization, a process dependent on the SDF-1/CXCR4 axis that enabled targeted recruitment of progenitor cells to bone repair sites. Furthermore, the pronounced bone-repairing effects of F-VGC correlated with its promotion of BMSC differentiation (Figure S5). Previous studies had demonstrated that GM-CSF directly upregulated key osteogenic genes such as *Runx2* and alkaline phosphatase (*ALP*) [12]. Collectively, these mechanisms suggested that actively modulating chemotaxis and osteogenesis-related signaling pathways can systematically enhance the recruitment and functional differentiation of cells required for bone repair. The observed increase in OPN expression in this study might represent one of the ultimate functional outcomes driven by such systemic cellular events.

### Repair effect of VGC on full-thickness skin defects in mice

**Figure 8:**
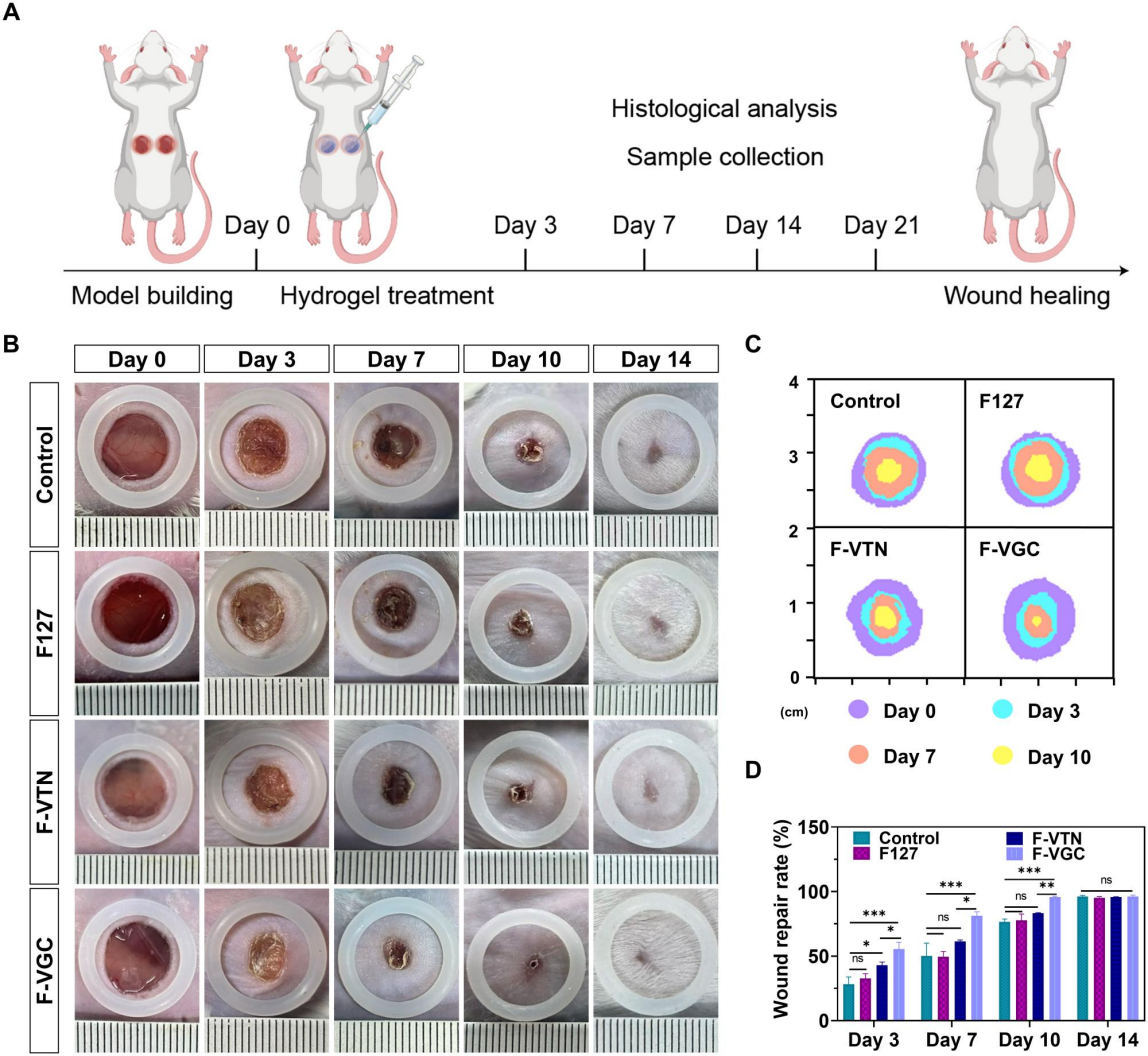
Dynamic observation and quantitative analysis of the skin wound healing process. (**A**) Schematic of the mouse full-thickness skin wound model. (**B**) Representative macroscopic images of wounds at the indicated time points; (**C**) Schematic diagram of wound area tracking; (**D**) Quantification of wound closure rates indicates that F-VGC significantly accelerates healing on days 3, 7, and 10 (*P* < 0.001), *n* = 3, *P < 0.05, ***P < 0.001. Data are presented as mean **±** standard deviation.

Building on the demonstrated efficacy of F-VGC in promoting bone regeneration, we next evaluated its therapeutic potential in soft tissue repair and immunomodulation using a mouse full-thickness excisional skin wound model (Figure 8A).

Morphological assessment revealed better wound closure in the F-VGC group compared to the control, F127 and F-VTN groups (Figure 8B). By Day 14, F-VGC-treated wounds achieved near-complete re-epithelialization. Dynamic wound area tracking showed a higher contraction rate in the F-VGC group (Figure 8C). Quantitative healing rates demonstrated that F-VGC significantly accelerated repair throughout the process (Figure 8D). On Day 3, the healing rate in the F-VGC group (55.41 ± 5.27%) markedly exceeded that in both the control (28.19 ± 5.63%; ***P < 0.001) and F-VTN groups (43.01 ± 2.44%; *P < 0.05). This accelerated healing persisted on Day 7, with the F-VGC group (81.12 ± 3.17%) remaining significantly ahead of the control (50.17 ± 9.89%; ***P < 0.001). The F-VGC group maintained the highest healing rate on Day 10 (95.78 ± 0.65%; ***P < 0.001). By Day 14, healing rates had converged near completion across all groups, with no significant differences. These results demonstrated that the F-VGC hydrogel consistently promoted wound healing, particularly during the critical early and middle phases, offering a significant advantage over formulations containing the individual components alone.

**Figure 9:**
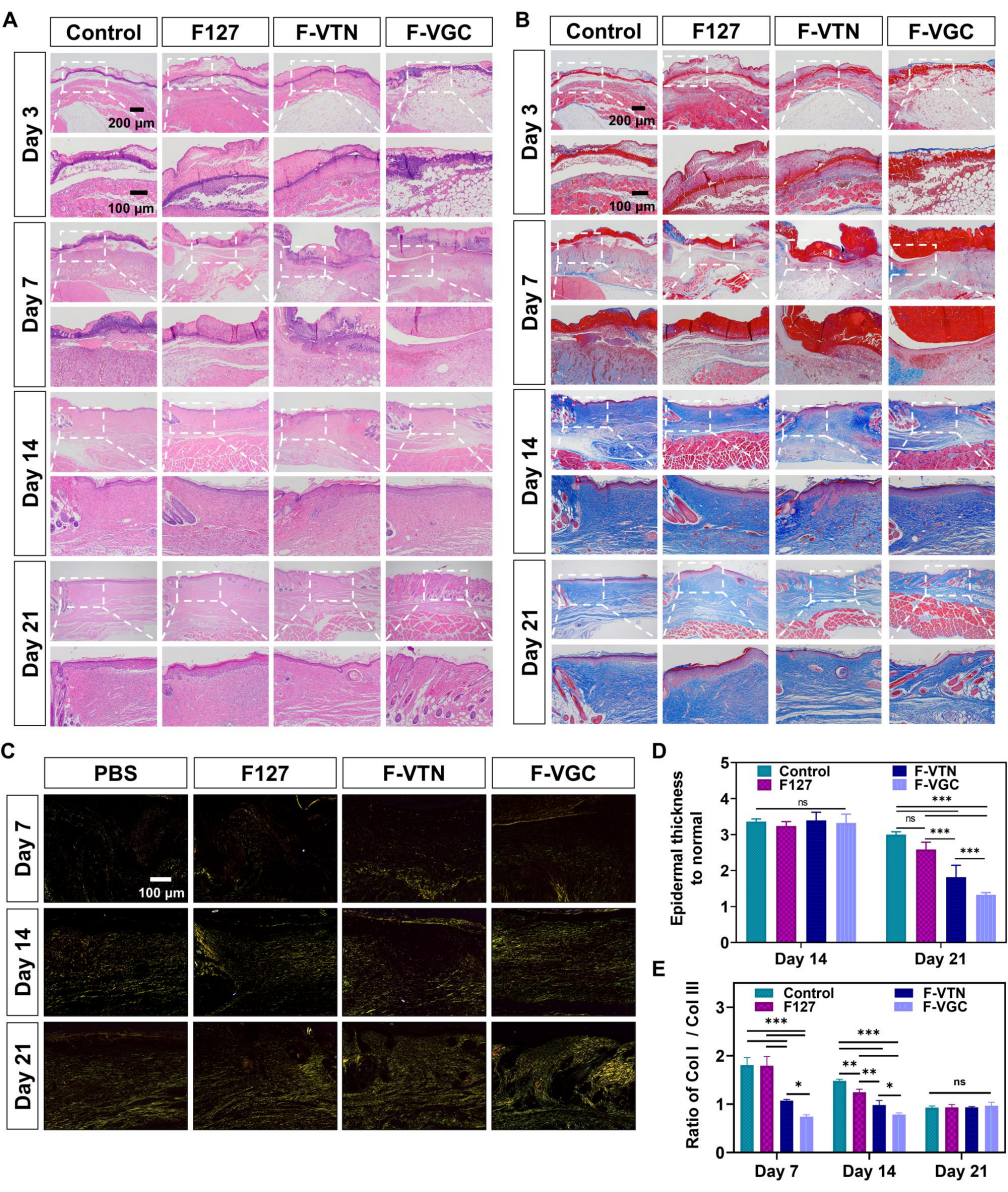
Histological analysis of skin regeneration and collagen remodeling. (**A**) H&E and (**B**) MTC staining of wound tissue on postoperative Days 3, 7, 14 and 21. (**C**) Polarized light micrographs of picrosirius red-stained on Days 7, 14 and 21, distinguishing type I (orange-red) and type III (green) collagen. (**D**) Quantitative ratio of neo-epidermal to native epidermal thickness on Days 14 and 21. The ratio of neo-epidermal thickness to native epidermal thickness in the F-VGC group was closer to one compared to the other groups, suggesting that its thickness more closely resembled that of the native epidermis. (**E**) Quantitative ratio of type I to type III collagen area. The ratio of Col I to Col III was the lowest in the F-VGC group, suggesting that Col III constituted a higher proportion in the newly formed skin of the F-VGC group during the repair process, until healing at 21 days post-injury when the ratios in all groups showed no statistically significant difference. Data are presented as mean ± standard deviation (*n* = 3); *P < 0.05, **P < 0.01, ***P < 0.001.

H&E staining was performed to assess newly formed skin tissue on postoperative Days 3, 7, 14 and 21 (Figure 9A). No epidermal regeneration was observed in any group by Day 3. By Day 7, the F-VGC group showed greater epidermal regeneration. Complete epidermal layers had formed in all groups by Day 14. The ratio of newly formed epidermal thickness to native epidermal thickness was similar among all groups at this time point (F-VGC: 3.32 ± 0.25; F-VTN: 3.40 ± 0.22; F127: 3.24 ± 0.12; control: 3.36 ± 0.07). By Day 21, this ratio was significantly lower in both the F-VGC (1.33 ± 0.06) and F-VTN (1.82 ± 0.33) groups compared to the control (2.99 ± 0.82; ***P < 0.001) and F127 (2.59 ± 0.20; ***P < 0.001) groups. Notably, the ratio in the F-VGC group was closest to 1, indicating that its epidermis thickness most closely approximated that of native skin and was significantly better restored than in the F-VTN group (Figure 9D).

MTC and Sirius red staining evaluated collagen deposition and maturation during repair (Figure 9B and C). On Day 7, collagen formation and angiogenesis were minimal in all groups. By Day 14, collagen remained sparse and disorganized in the PBS and F127 groups. The F-VTN group showed increased yet irregular deposition. In contrast, the F-VGC group exhibited denser, more extensive and well-organized collagen fibers, approximating native skin architecture. Concurrently, the F-VGC group showed notably strengthened angiogenesis phenomenon, with vessel numbers being more numerous and vessels being more mature that have clear lumens when compared with other groups. On Day 21, the F-VGC group presented the thickest, most plentiful and best-aligned collagen bundles, and at the same time, a mature vascular network.

Sirius red staining indicated that, by the endpoint, the F-VGC group exhibited the most abundant and regularly arranged collagen deposition situation. Quantitative analysis of collagen I/III ratio provided understanding toward remodeling dynamics (Figure 9E). This ratio was significantly lower in F-VGC and F-VTN groups on Days 7 and 14 compared to the control groups (***P < 0.001), indicating a repair phase dominated by type III collagen for rapid defect filling. By Day 21, the ratios in all groups converged within a stable, physiological range, marking the transition into balanced collagen metabolism. The final ratio in the F-VGC group most closely approximated that of normal tissue. In summary, the F-VGC hydrogel promoted wound healing by accelerating collagen deposition and organization, which resulted in a regenerated dermal microstructure that closely mimicked native skin, thereby restoring mechanical properties and function.

**Figure 10:**
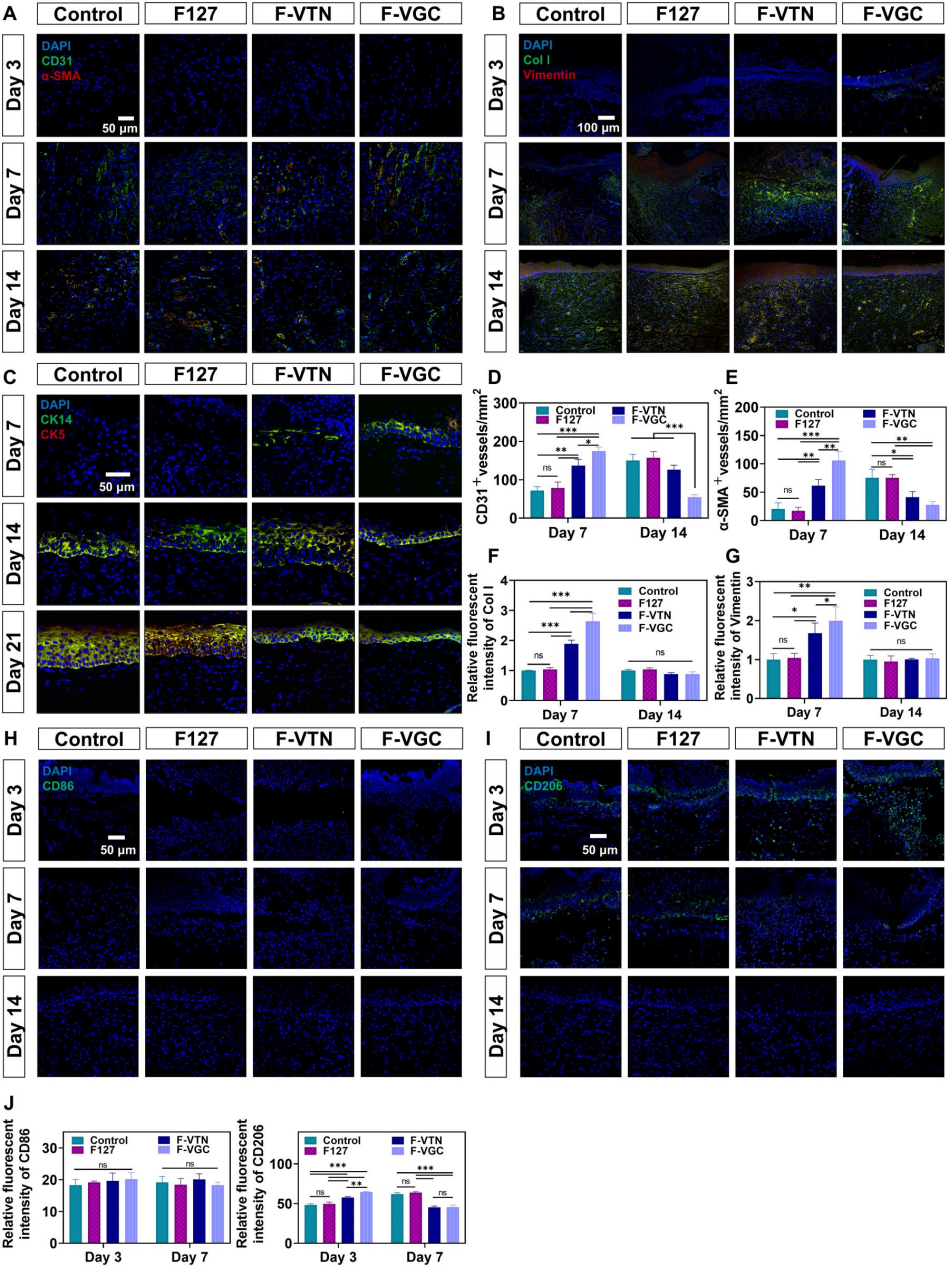
Immunofluorescence analysis of key processes during skin wound healing. (**A**) Co-localization of α-SMA (red) and CD31 (green) on Days 3, 7 and 14 post- wounding (nuclei: DAPI/blue). (**B**) Expression of col I (green) and vimentin (red) in the newly formed skin on Days 3, 7 and 14. (**C**) Expression of cytokeratin 14 (CK14, green) and cytokeratin 5 (CK5, red) in the newly formed epidermis on Days 7, 14 and 2 1, indicating re-epithelialization. Quantitative analysis of the positive area for (**D**) CD31 and (**E**) α-SMA. At 7 days post-operation, the number of CD31^+^ and α-SMA^+^ vessels in the F-VGC group was significantly higher than that in the other groups, at 14 days post-operation, by which time repair was essentially complete, it had decreased. Quantification of the relative fluorescence intensity for (**F**) Col I and (**G**) vimentin. The level of Col I in the F-VGC group was significantly higher than in the other groups at 7 days post-operation (*P* < 0.001), while no significant difference was observed among the groups at 14 days post-operation (*P* > 0.05). The level of vimentin in the F-VGC group was also significantly higher than in the other groups at 7 days post-operation. Representative images showing the distribution of (H) CD86^+^ and (**I**) CD206^+^ cells. (**J**) Quantification of CD86 and CD206 fluorescence intensity. At 3 days post-operation, the level of CD206 in the F-VGC group was significantly higher than in the F-VTN group (*P* < 0.01), while no statistically significant difference was observed between the two groups at 7 days post-operation (*P* > 0.05). Data are presented as mean ± standard deviation (*n* = 3); *P < 0.05, **P < 0.01, ***P < 0.001.

Neovascularization was assessed using CD31 and α-SMA immunofluorescence staining (Figure 10A). No neovascularization was observed in any group by postoperative Day 3. By Day 7, CD31-positive vascular density (Figure 10D) in both F-VGC (174.7 ± 10.50, ***P < 0.001) and F-VTN (136.7 ± 15.82, **P < 0.01) groups was significantly higher than that in the control group. Consistent with this, analysis of α-SMA (Figure 10E) revealed that the number of mature vessels in F-VGC (106.0 ± 15.72, ***P < 0.001) and F-VTN (61.67 ± 10.50, **P < 0.01) groups was also significantly higher than that in the control group. By Day 14, CD31-positive vascular density revealed that the F-VGC group (54.67 ± 6.35, ***P < 0.001) was significantly lower than the control group (150.3 ± 15.82), whilst the F-VTN group and F127 hydrogel group showed no significant difference from the control group. Analysis of α-SMA-positive vessels similarly revealed that both the F-VGC group (27.67 ± 5.77, **P < 0.01) and the F-VTN group (41.00 ± 10.00, *P < 0.05) were significantly lower than the control group. These findings indicated that F-VGC hydrogel significantly promoted angiogenesis during the early repair phase while accelerating vascular maturation and remodeling processes in the later stages.

To evaluate the remodeling of the extracellular matrix during skin tissue repair, this study quantitatively analyzed the expression of Col I and vimentin by immunofluorescence staining, with results presented as relative fluorescence intensity. As shown in Figure 10B, Col I was detectable in newly formed skin tissue across all groups by Day 7. Quantitative analysis revealed that the relative fluorescence intensity of Col I was significantly higher in the F-VGC group (2.64 ± 0.27, ***P < 0.001) and the F-VTN group (1.89 ± 0.12, ***P < 0.001) compared to the control and F127 groups (Figure 10F). By Day 14, no significant differences in Col I intensity were observed, indicating stabilization of matrix remodeling. Analysis of vimentin expression showed that on Day 7, its intensity was significantly higher in the F-VGC group (1.99 ± 0.36; **P < 0.01) and the F-VTN group (1.68 ± 0.26; *P < 0.05) than in the control (1.00 ± 0.15) and F127 (1.04 ± 0.12) groups (Figure 10G). By Day 14, vimentin expression in the F-VGC group had decreased significantly compared to Day 7, and no significant differences were found among groups, suggesting the re-epithelialization process was largely complete (Figure 10G). These findings indicated that the F-VGC hydrogel significantly promoted early deposition of Col I and expression of vimentin during repair.

Re-epithelialization, which is a core process inside wound healing that has relation with keratinocyte activation, migration and differentiation, was dynamically evaluated through keratin expression (Figure 10C). On Day 7, no new epidermis was observed in the control or F127 groups. Although epidermal growth was evident in the F-VTN group, the structure remained incomplete. In contrast, the F-VGC group had already formed a continuous epidermis. By Day 14, keratin expression remained low in the control group. Both the F127 and F-VTN groups exhibited epidermal thickening. Notably, the F-VGC group displayed fully differentiated keratinocytes, achieving complete wound closure and restored epidermal thickness. On Day 21, the control group still showed incomplete differentiation, while the F127 group had entered the remodeling phase. The F-VTN group showed complete healing with normalized thickness.

To evaluate the role of macrophage polarization in skin repair, we quantitatively analyzed the expression of the pro-inflammatory marker CD86 and the anti-inflammatory marker CD206 by immunofluorescence (Figure 10H and I). Quantitative analysis (Figure 10J) showed no significant differences in CD86 expression among groups. On Day 3, the relative fluorescence intensity of CD206 in the F-VGC group (64.59 ± 0.93) was significantly higher than that in the F-VTN group (57.70 ± 1.05; **P < 0.01), the control group (48.11 ± 1.63; ***P < 0.001) and the F127 group (49.44 ± 2.28; ***P < 0.001). By Day 7, the CD206 level in the F-VGC group (45.40 ± 2.62; ***P < 0.001) was significantly lower than that in the control (61.97 ± 1.70) and F127 (63.76 ± 1.19) groups but showed no significant difference compared to the F-VTN group (45.38 ± 1.52).

These results demonstrated that the F-VGC hydrogel effectively promoted the structural and functional repair of skin *in vivo*. This effectiveness relied on several coordinated mechanisms. First, it had a powerful early-stage angiogenic ability, as shown by a notable rise of CD31^+^ and α-SMA^+^ vessels on Day 7 (Figure 10). Second, it possessed immunomodulatory capacity, promoting macrophage polarization toward the M2 phenotype (Figures 4E and 10J). Third, it directly activated and regulated key repair-related cells, including dermal fibroblasts and epidermal keratinocytes.

*In vitro* experiment indicated that VTN, which was a core component of F-VGC, directly promoted the proliferation and migration of HDFs, and this effect was further strengthened by GM-CSF (Figure 5). This was consistent with existing literature showing that VTN promoted fibroblast activation and matrix deposition via integrin-mediated pathways [33]. *In vivo*, this regulatory effect was directly supported by the strongest expression of collagen I and vimentin in the F-VGC group on Day 7, confirming robust activation of fibroblast synthetic function (Figure 10B). The GM-CSF domain directly promoted the proliferation and migration of epidermal keratinocytes [34, 35], which likely accounts for the superior re-epithelialization observed in the F-VGC group, where a complete neo-epidermis formed earlier than in other groups. Furthermore, GM-CSF may further enhance these cellular responses by indirectly generating beneficial paracrine signals through immunomodulation [7].

Although this study was conducted in a normal wound healing model, the comprehensive benefits of the F-VGC system in angiogenesis, epithelial regeneration, collagen remodeling and immunomodulation provided a solid experimental basis for its potential application to complex wounds with pathological microenvironments, such as diabetic foot ulcers. In subsequent studies, we will employ diabetic wound and bone defect models, increase the sample size and progress from small to more clinically relevant large animal models, with the ultimate goal of facilitating the clinical translation of VGC hydrogels.

**Figure 11:**
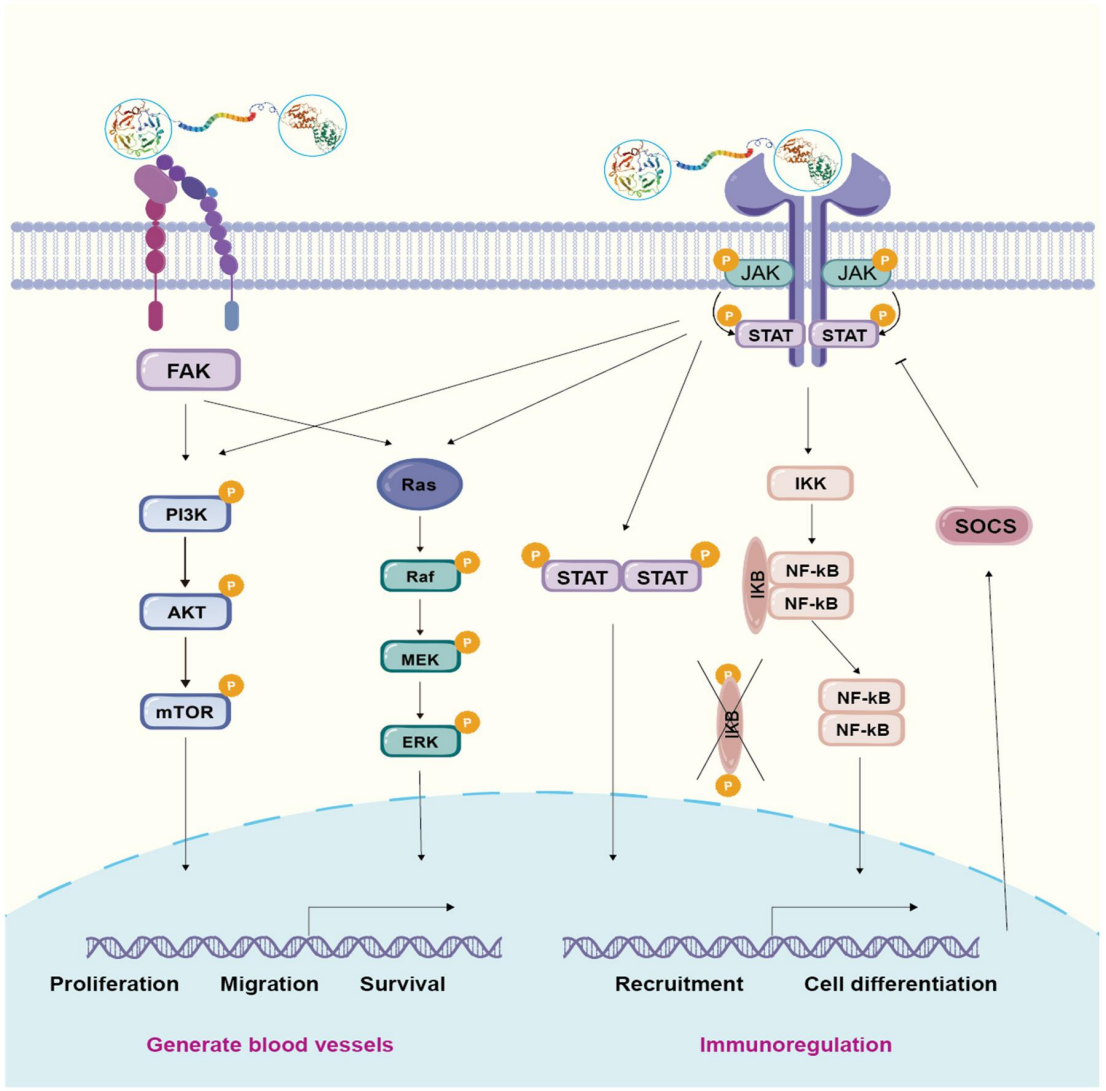
Schematic illustrating the core biological processes regulated by the VGC fusion protein. The VTN domain in VGC targets cell surface integrins, activates FAK, and initiates the PI3K/AKT/mTOR and Ras/Raf/MEK/ERK signaling pathways. The GM-CSF domain activates the JAK-STAT and NF-κB pathways. The diagram summarizes its coordinated effects on cell differentiation, migration, survival and immunomodulation. Created with BioGDP.com [24].

We designed and constructed a VTN-linker-GM-CSF fusion protein that promoted tissue repair through domain-mediated synergistic mechanisms (Figure 11). Based on prior literature, the VTN domain targeted cell surface integrins, activating FAK and initiating the PI3K/AKT/mTOR and Ras/Raf/MEK/ERK signaling pathways [29, 36–39], thereby promoting cell proliferation, migration, survival and angiogenesis. The GM-CSF domain, upon receptor binding, activated JAK-STAT and NF-κB pathways [40–43] to regulate immune cell recruitment, activation and differentiation. The signaling pathways of the VTN and GM-CSF domains converged and interacted, forming a synergistic regulatory network. Ultimately, through a recruitment-anchoring-activation mechanism, they cooperatively promoted angiogenesis and immune regulation in both temporal and spatial dimensions, offering a novel strategy for efficient *in situ* tissue repair.

## Conclusions

This study developed an innovative biomaterial system based on a VTN-GM-CSF (VGC) fusion protein. It effectively addressed the ‘recruitment without retention’ challenge in cytokine therapy through a synergistic ‘recruit-retain-activate’ mechanism. The VGC fusion protein significantly promoted the proliferation, migration and tube formation of vascular endothelial cells and spatiotemporally regulated the phenotypic transition of macrophages from the pro-inflammatory M1 to the pro-repair M2 state. It also enhanced the activity of other key repair cells, including fibroblasts and osteoblasts. When incorporated into a thermosensitive F127 hydrogel, the system accelerated bone regeneration in a rat cranial defect model by increasing the BV/TV and collagen deposition. In a full-thickness skin wound model, it promoted high-quality healing via early angiogenesis, collagen remodeling and re-epithelialization. By integrating VTN and GM-CSF, the fusion protein ultimately demonstrated dual functionality in promoting vascularization and immunomodulation for both hard and soft tissue repair. This work provided a novel design paradigm for developing multi-functional protein-based materials for *in situ* tissue regeneration.

## Supplementary Material

rbag100_Supplementary_Data
