# Supplementary material for: Vitronectin-GM-CSF fusion protein hydrogel with a recruitment-anchoring-activation strategy accelerates vascularized tissue regeneration
Source: Regen Biomater. 2026 May 23;13:rbag100. doi: 10.1093/rb/rbag100 (PMC13291906; doi:10.1093/rb/rbag100)
Supplement: rbag100_Supplementary_Data [file rbag100_Supplementary_Data.zip › Supplementary_Information-revised.docx]

**Vitronectin-GM-CSF Fusion Protein Hydrogel with a Recruitment-Anchoring-Activation Strategy Accelerates Vascularized Regeneration**

Jiake Zhang, Yuhan Xia, Xueliang Peng, Wenjing Li, Yuzhen Zhao, Fulin Chen, Zhuoyue Chen*

Provincial Key Laboratory of Biotechnology of Shaanxi, Key Laboratory of Resource Biology and Modern Biotechnology in Western China, Faculty of Life Sciences, Northwest University, 229 North Taibai Road, Xi’an, Shaanxi Province, 710069, China

^*^ Corresponding author.

*Email address*: [zychen@nwu.edu.cn](mailto:zychen@nwu.edu.cn) (Z. Chen)


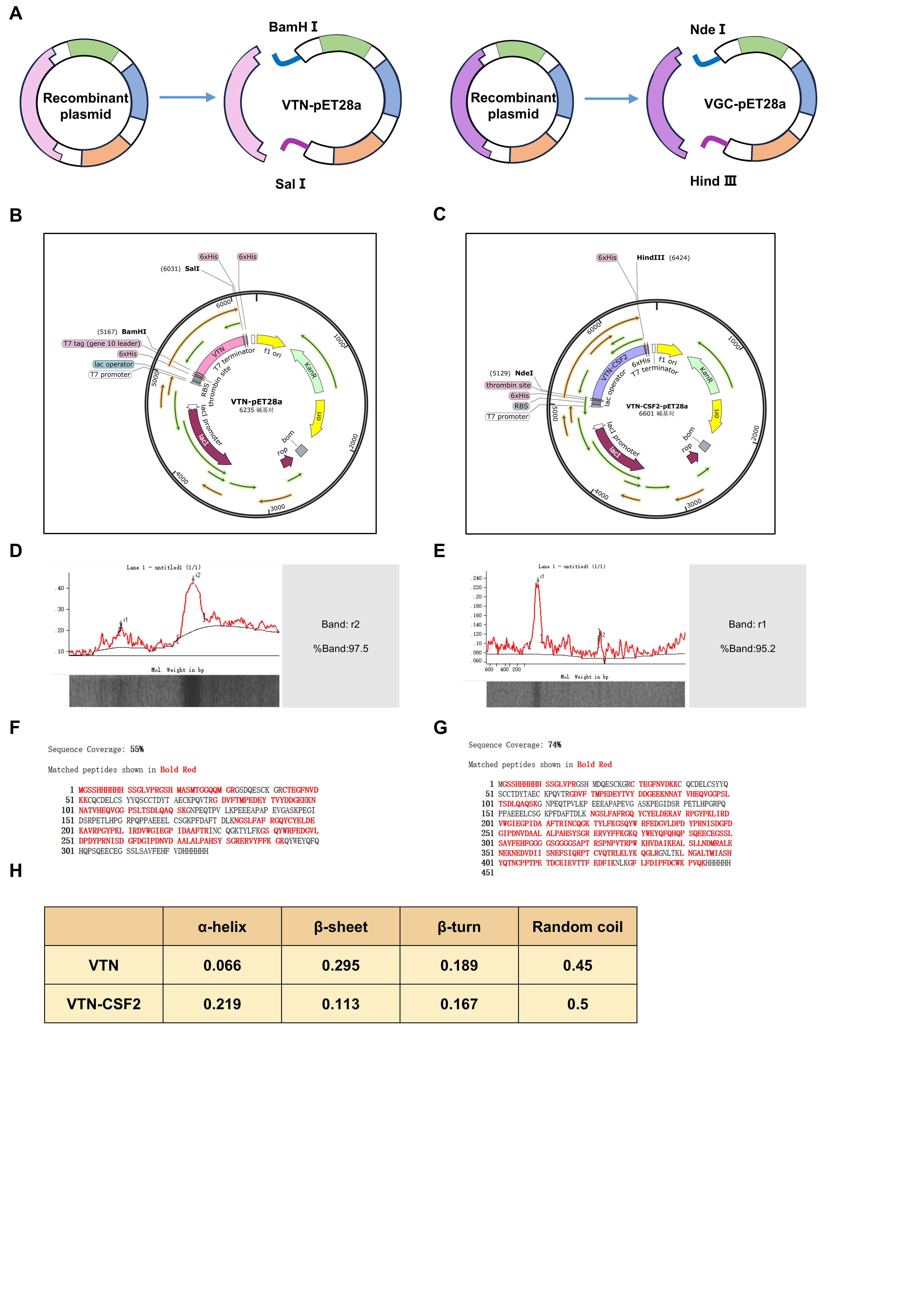


**Figure S1. Plasmid construction, expression, purification, and structural characterization of the recombinant proteins VTN and VGC.** (A) Schematic of the plasmid synthesis process for VTN and VGC. Plasmid map of the (B) VTN construct and (C) VGC construct. (D) Purity analysis of the VTN protein and (E) VGC protein. Mass spectrometry analysis of the (F) VTN protein and (G) VGC protein. (H) Percentages of α-helix, β-sheet, β-turn, and random coil structures in the VTN and VGC proteins.


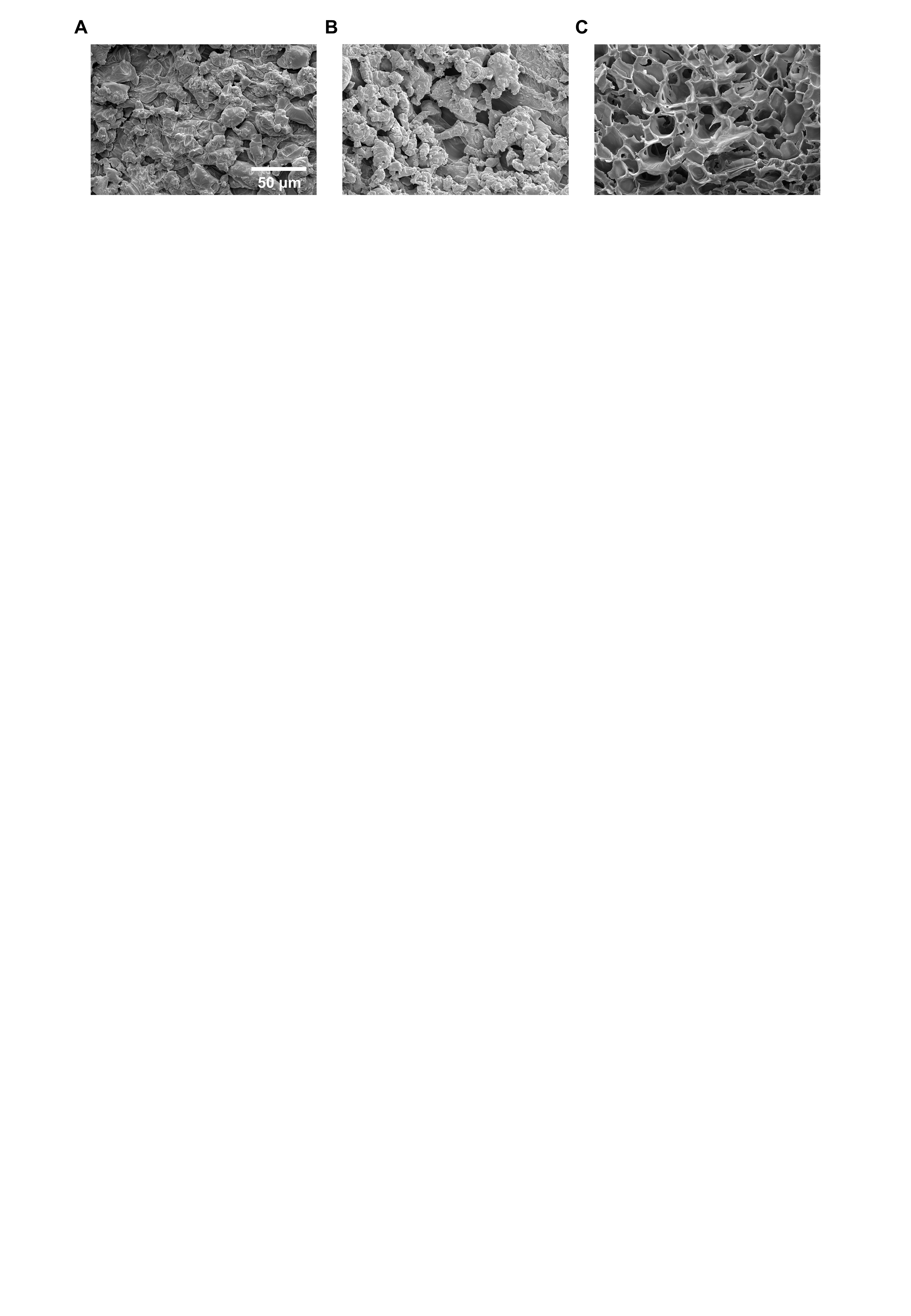


**Figure S2. Microstructure of F127 hydrogels at varying concentrations.** Representative SEM images of (A) 10%, (B) 20%, and (C) 30% F127 hydrogels. The 10% hydrogel exhibits a loose, irregular pore structure. Pore connectivity and structural uniformity improve in the 20% hydrogel. The 30% hydrogel forms a dense, regular 3D network with optimized pore morphology.


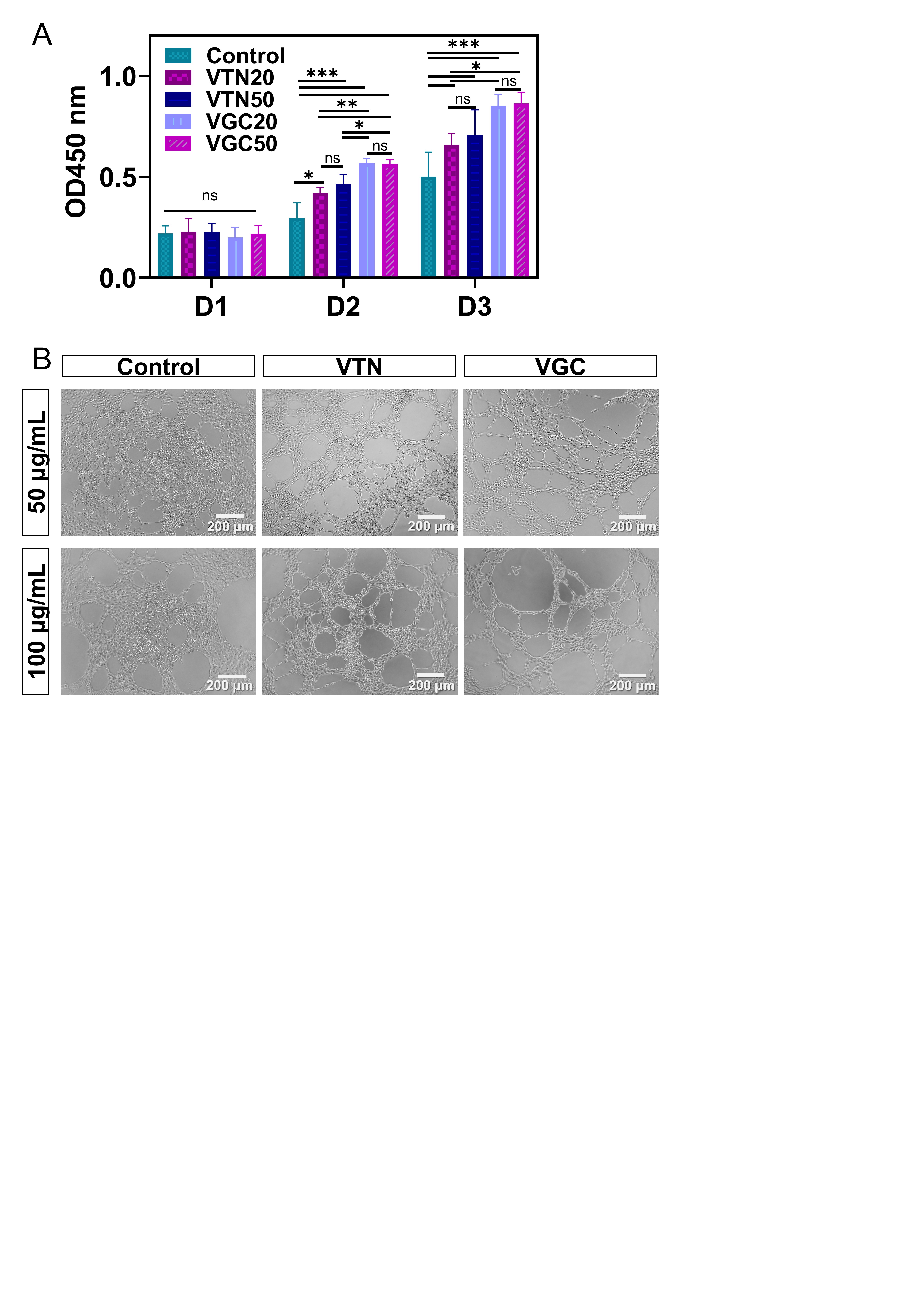


**Figure S3. Dose-dependent effects of VTN and VGC on cell proliferation and tubule formation.** (A) Cell proliferation assessed by OD450 measurement over 3 days post-treatment. No significant difference was observed between 20 μg/mL and 50 μg/mL (P > 0.05, n=6), therefore, the lower concentration (20 μg/mL) was selected for subsequent experiments. (B) Representative images showing morphologic effects of VTN and VGC at 50 and 100 μg/mL. All data are presented as mean ± standard deviation. *P<0.05, **P<0.01, ***P<0.001.


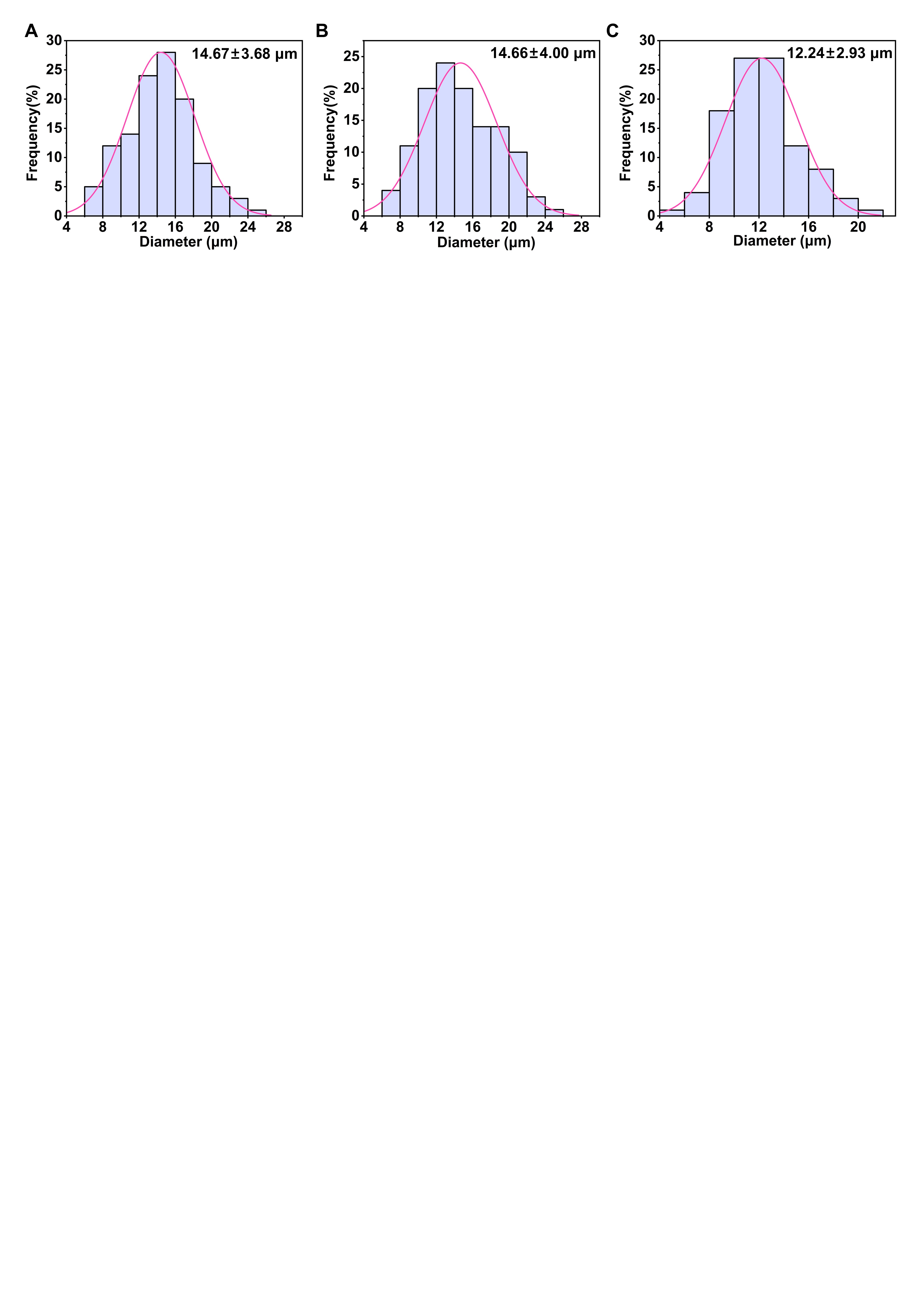


**Figure S4. Pore size distribution statistics for composite hydrogels.** Pore size distribution histograms of F127 hydrogel (A) VTN/F127 hydrogel (B) and VTN-CSF2/F127 hydrogel (C) respectively (n=33 pores counted per sample, N=3 independent samples).


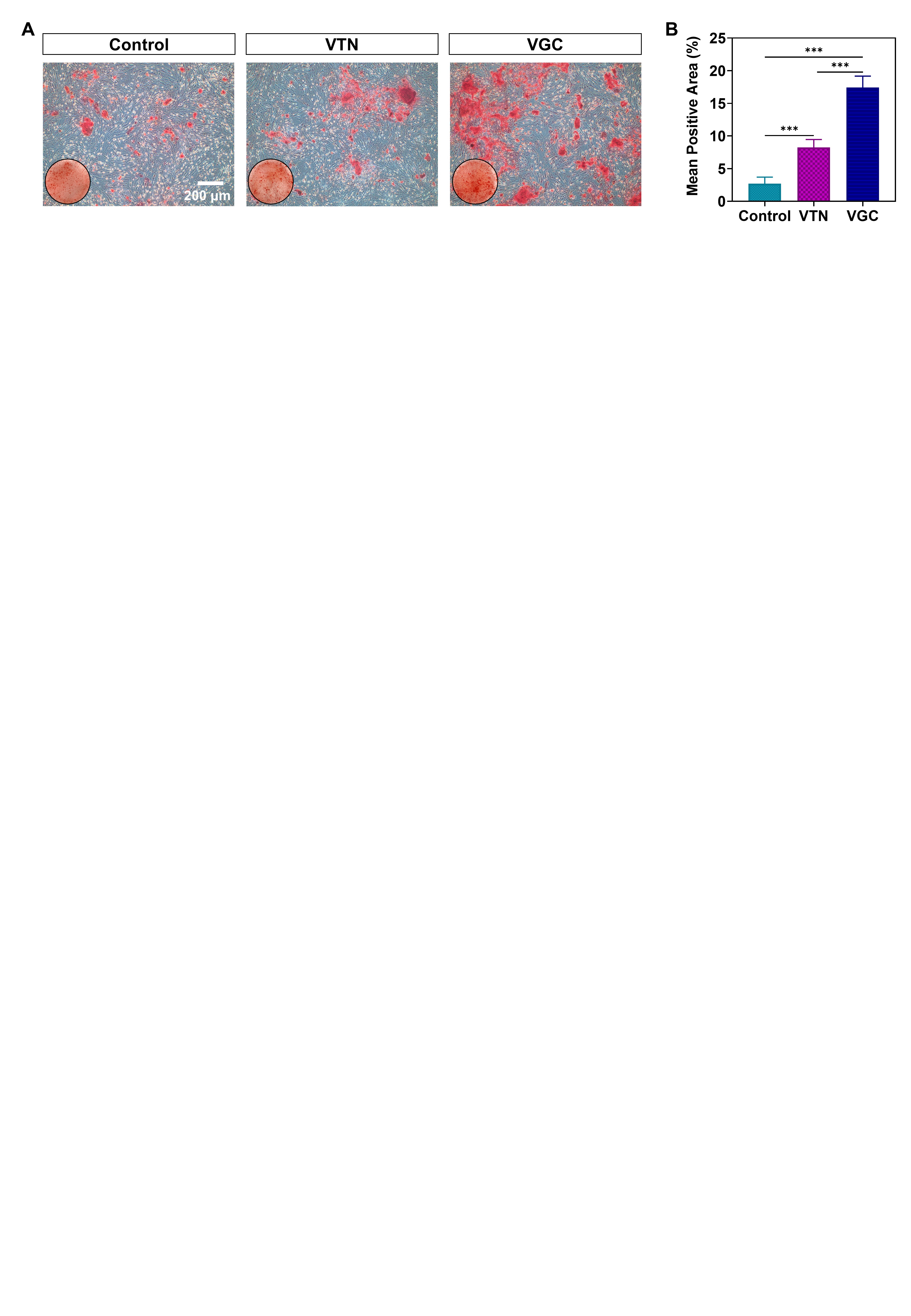


**Figure S5. Effects of VGC proteins on the osteogenic differentiation of BMSCs.** (A) ARS staining images of bone marrow mesenchymal stem cells after 18 days of treatment with VTN or VGC (20 μg/mL); (B) Quantitative analysis of ARS staining, n=6, **P<0.01, ***P<0.001; values are expressed as mean ± standard deviation.


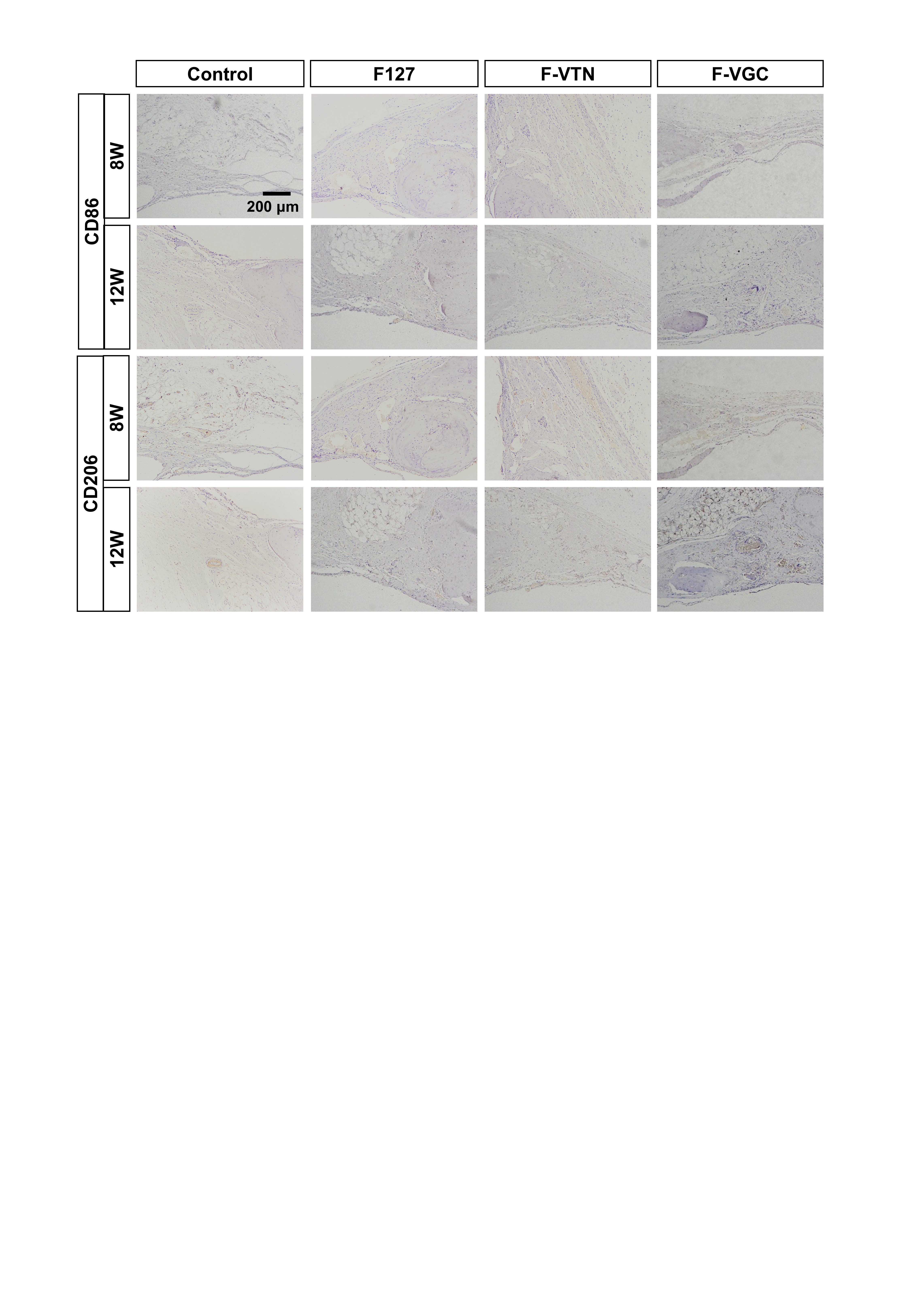


**Figure S6. Assessment of macrophage polarization in the bone defect model via immunohistochemistry.** The presence and distribution of M1 (CD86) and M2 (CD206) macrophages were evaluated in the newly formed osseous tissue at the 8 and 12 week time points post-implantation.

Table S1: List of Primary and Secondary Antibodies Used in the Study

| Antibody | Manufacturer |
| --- | --- |
| OPN（rabbit，Cat No.22952-1-AP） | Protein Tech |
| Col I（rabbit，Cat No.AB138492） | Abcam |
| CD31（rabbit，Cat No. 28083-1-AP） | Protein Tech |
| α-SMA（mouse，Cat No. 67735-1-Ig） | Protein Tech |
| Vimentin（mouse，Cat No. 60330-1-Ig） | Protein Tech |
| CK5（rabbit，Cat No. 26411-1-AP） | Protein Tech |
| CK14（mouse，Cat No. 10143-1-AP） | Protein Tech |
| CoraLite488-conjugated Goat Anti-Rabbit IgG(H+L)( Cat No. SA00013-2) | Protein Tech |
| CoraLite594-conjugated Goat Anti-Mouse IgG(H+L)( Cat No. SA00013-3) | Protein Tech |

Table S2: Amino acid sequence of VTN and VGC proteins

| **Amino acid sequence of VTN** |
| --- |
| MGSSHHHHHHSSGLVPRGSHMASMTGGQQMGRGSDQESCKGRCTEGFNVDKKCQCDELCSYYQSCCTDYTAECKPQVTRGDVFTMPEDEYTVYDDGEEKNNATVHEQVGGPSLTSDLQAQSKGNPEQTPVLKPEEEAPAPEVGASKPEGIDSRPETLHPGRPQPPAEEELCSGKPFDAFTDLKNGSLFAFRGQYCYELDEKAVRPGYPKLIRDVWGIEGPIDAAFTRINCQGKTYLFKGSQYWRFEDGVLDPDYPRNISDGFDGIPDNVDAALALPAHSYSGRERVYFFKGKQYWEYQFQHQPSQEECEGSSLSAVFEHFVDHHHHHH* |
| **Amino acid sequence of VGC** |
| MGSSHHHHHHSSGLVPRGSHMDQESCKGRCTEGFNVDKKCQCDELCSYYQSCCTDYTAECKPQVTRGDVFTMPEDEYTVYDDGEEKNNATVHEQVGGPSLTSDLQAQSKGNPEQTPVLKPEEEAPAPEVGASKPEGIDSRPETLHPGRPQPPAEEELCSGKPFDAFTDLKNGSLFAFRGQYCYELDEKAVRPGYPKLIRDVWGIEGPIDAAFTRINCQGKTYLFKGSQYWRFEDGVLDPDYPRNISDGFDGIPDNVDAALALPAHSYSGRERVYFFKGKQYWEYQFQHQPSQEECEGSSLSAVFEHFGGGGSGGGGSAPTRSPNPVTRPWKHVDAIKEALSLLNDMRALENEKNEDVDIISNEFSIQRPTCVQTRLKLYKQGLRGNLTKLNGALTMIASHYQTNCPPTPETDCEIEVTTFEDFIKNLKGFLFDIPFDCWKPVQKHHHHHH* |

Flexible linker: GGGGSGGGGS.
