# Supplementary material for: Vitronectin-GM-CSF fusion protein hydrogel with a recruitment-anchoring-activation strategy accelerates vascularized tissue regeneration
Source: Regen Biomater. 2026 May 23;13:rbag100. doi: 10.1093/rb/rbag100 (PMC13291906; doi:10.1093/rb/rbag100)
Supplement: rbag100_Supplementary_Data [file rbag100_Supplementary_Data.zip › Response.docx]

**Re: Manuscript ID: RB-2026-124.R1**

Dear Editor,

Thank you very much for efficiently handling our manuscript. All items listed below have been carefully revised point by point.

Guidelines for figures: Please carefully check all figures to avoid reusing images or incorrect labeling, as such issues may be treated as academic misconduct.

Reply: Done.

Insert figures at the point of first mention in the main text and provide corresponding figure legends.

Reply: Done.

Except for the graphical abstract, all figures should be numbered as Figure 1, 2, 3, etc., including scheme diagrams. Do not use a separate numbering system like Scheme 1.

Reply: Done.

For primary image titles, use A, B, C; for secondary image titles, use a, b, c, without parentheses.

Reply: Done.

Ensure consistency across all figures and the entire text in terms of time units.

Reply: Done.

Abbreviations or full forms should be used properly and consistently. Except for the percent sign (%) and temperature symbols (℃ or ℉), a space should be added after all numbers before the unit. This includes numbers within scale bars, which also require a space before the unit.

Reply: Completed.

Please note that the manuscript you are submitting now will be published in advance. Please check it carefully, remove all revision marks and highlighting, and resubmit the final version in Microsoft Word format for Advance Access publication.

Reply: Completed.

Best regards,

Associate professor

Faculty of Life Science

Northwest University, Xi'an, Shaanxi, China.

E-mail: [zychen@nwu.edu.cn](mailto:zychen@nwu.edu.cn)
